# Antibacterial Activity of Selected Essential Oil Components and Their Derivatives: A Review

**DOI:** 10.3390/antibiotics14010068

**Published:** 2025-01-10

**Authors:** Vuyolwethu Khwaza, Blessing A. Aderibigbe

**Affiliations:** Department of Chemistry, Faculty of Science and Agriculture, University of Fort Hare, Alice Campus, Alice 5700, South Africa

**Keywords:** antibacterial activity, essential oils, natural products, monoterpenes, phenylpropanoids, derivatives/analogs

## Abstract

Essential oils (EOs) are gaining ground and have been intensively studied due to their widespread use in the pharmaceutical, food, and cosmetics industries. The essential components of EOs have been recognized for diverse therapeutic activities and have gained significant attention for their potential antibacterial activities. Despite the popularity of EOs and potent biological properties, their bioactive components and their derivatives are still not comprehensively characterized. This review explores the antibacterial efficacy of selected EO components and their derivatives, focusing on monoterpenes chosen (i.e., carvacrol, menthol, and thymol) and phenylpropanoids (i.e., cinnamaldehyde and eugenol). Furthermore, this review highlights recent advancements in developing derivatives of these EO components, which have shown improved antibacterial activity with reduced toxicity. By summarizing recent studies, this review reveals the potential of these natural compounds and their derivatives as promising candidates for pharmaceuticals, food preservation, and as alternatives to synthetic antibiotics in combating bacterial resistance.

## 1. Introduction

In an era where the increasing prevalence of antibiotic resistance poses a significant challenge to global healthcare [1,2,3], the search for alternative and effective antibacterial agents has become more critical than ever. In 2019, it was estimated to have directly caused 1.27 million deaths globally and contributed to 4.95 million deaths [4]. The rise in antibiotic resistance is diminishing the effectiveness of common antibiotics against widespread bacterial infections [4]. The 2022 World Health Organization (WHO) Global Antimicrobial Resistance and Use Surveillance System (GLASS) report highlights alarming resistance rates [2,4]. The median rates reported in 76 countries were 42% for third-generation cephalosporin-resistant *Escherichia coli* and 35% for methicillin-resistant *Staphylococcus aureus* (MRSA) [4]. Regarding urinary tract infections caused by *E. coli*, one in five cases exhibited reduced susceptibility to standard antibiotics like ampicillin, co-trimoxazole, and fluoroquinolones in 2020 [4]. Resistance is also increasing in bacteria like *Klebsiella pneumoniae*, leading to the greater use of last-resort antibiotics like carbapenems, for which resistance is also being observed [2,4]. This raises the risk of infections that cannot be effectively treated.

EO components extracted from aromatic plants have attracted considerable attention due to their diverse biological activities, particularly their notable antibacterial properties [5,6]. These components are complex mixtures of bioactive compounds, with phenolic monoterpenoids such as carvacrol, thymol, eugenol, and cinnamaldehyde standing out as key contributors to their antimicrobial effects [7]. These EO components have been proven to offer a plethora of pharmacological activities, including anticancer [8], antibacterial [9], anti-arthritis [10], antiviral [11], anti-inflammatory [12,13], antifungal, and antioxidant activities [14]. The pharmacological properties are associated with the bioactive compounds in EOs, as well as the structural arrangement and functional groups of these compounds. EOs derived from natural plants can also be used in the food industry to prolong the shelf life of food products and manage food spoilage [15,16].

**Figure 1:**
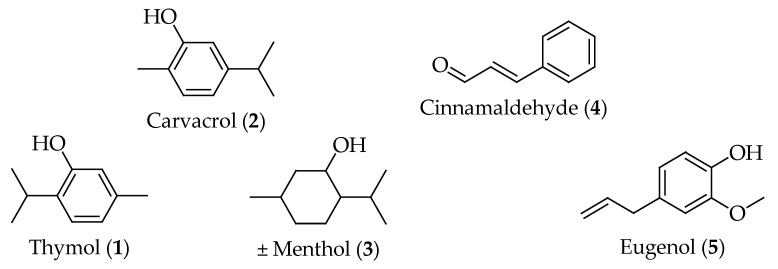
Chemical structures of the selected EO components.

The antibacterial efficacy of these EO components is primarily attributed to their ability to disrupt bacterial membranes [17], inhibit enzymatic processes [18], and prevent biofilm formation [19], among other mechanisms. Thymol, menthol, and carvacrol from the monoterpene group, along with eugenol and cinnamaldehyde from the phenylpropanoids group (in Figure 1), are among the most promising EO components with antibacterial activity. These bioactive compounds have garnered significant attention due to their broad-spectrum antibacterial properties, the potential for low toxicity, and the ability to target multiple bacterial pathways. The antimicrobial effects of these compounds are closely related to their physicochemical properties, such as lipophilicity, partition coefficient, and hydrogen bonding characteristics [20]. The chemical modifications of these natural compounds and their ability to produce derivatives have further enhanced their antibacterial potency and broadened their spectrum of activity. These derivatives often exhibit improved stability, solubility, and bioavailability, making them promising candidates for antibacterial drug development [21].

This review provides a detailed exploration of the antibacterial potential of the selected EO components and their derivatives. It examines their mechanisms of action and structure–activity relationships. Additionally, it highlights recent advancements in the synthesis and evaluation of these compounds, emphasizing their potential as scaffolds for novel antibacterial agents. By synthesizing current research findings, this review aims to shed light on the critical role of EO components in the ongoing quest for innovative strategies to combat bacterial infections.

## 2. Antibacterial Activity of the Selected EO Components

### 2.1. Monoterpenes

Monoterpenes, a class of terpenoids consisting of two isoprene units [22], are found in EOs of many aromatic plants such as thyme, eucalyptus, peppermint, etc. [23]. They exhibit various biological activities, including antimicrobial, anti-inflammatory, and antioxidant properties. Key monoterpenes, such as thymol, carvacrol, and menthol, have been extensively studied for their antibacterial properties, demonstrating efficacy against both Gram-positive and Gram-negative bacteria [5,24,25]. Their mechanisms of action are diverse, often involving disruption of the bacterial cell membrane [17], interference with enzymatic activity [18], and the inhibition of biofilm formation [19], making them versatile agents in the fight against bacterial infections.

#### 2.1.1. Thymol and Carvacrol

Thymol 1 (Figure 1), a phenolic monoterpene compound naturally found in EOs of various plant species, like *Thymus vulgaris* L., etc. (Table 1), exhibits a range of pharmacological activities. Thymol’s antibacterial mechanism involves compromising the integrity of the cell membrane, causing the leakage of intracellular materials, and ultimately resulting in bacterial cell death [26]. A recent study by Lui et al. [16] demonstrated that *Thymus vulgaris* L. EO and thymol possess strong antibacterial and antibiofilm activities against *Pseudomonas aeruginosa*. It can cause irreversible cell membrane damage and significant nucleic acid leakage. Additionally, thymol induces ROS accumulation and deoxyribonucleic acid (DNA) damage through intercalation, leading to cell death. Thymol also possesses strong antibacterial properties against drug-resistant *Streptococcus iniae*. Its potential antibacterial mechanisms include disrupting cell membrane and cell wall integrity, affecting normal binary division, causing nutrient limitation, inhibiting metal ion transport, interfering with nucleotide biosynthesis and DNA repair, and disrupting transcriptional pathways. Furthermore, in vivo studies have indicated that thymol can reduce cumulative mortality and bacterial load in tissues of *S. iniae*-infected channel catfish while significantly enhancing the activity of non-specific immune enzymes in serum. Another study revealed the antimicrobial mechanism of thymol against *Enterobacter sakazakii*. The minimum inhibitory concentration (MIC) of thymol for *E. sakazakii* BNCC 186088 was found to be 1.25 mg/mL. After undergoing treatment with thymol, cell membrane depolarization reduced intracellular ATP levels, and lower intracellular pH levels were observed, indicating compromised cell membranes and disrupted intracellular homeostasis [27]. Wang et al. [28] discovered that thymol compromises the membrane integrity of *S. aureus*, allowing it to penetrate the bacterial cell and bind to the minor groove of the DNA, leading to the slight destabilization of the DNA’s secondary structure. In a study by Yin et al. [29], thymol exhibited significant antibacterial activity against *S. iniae*, with MIC and MBC values of 128 and 256 µg/mL, respectively. Thymol disrupts the bacterial cell wall and membrane, leading to the leakage of intracellular contents and interfering with energy metabolism, membrane transport, and DNA processes. In vivo experiments on channel catfish showed that thymol reduced mortality, lowered bacterial colonization in tissues, and boosted immune enzyme activities, highlighting its potential as a natural alternative to conventional antibiotics for treating *S. iniae* infections.

Carvacrol, a phenolic cyclic monoterpenoid isomer of thymol, is also found in the essential oils of several plant species (see Table 1). The predominant mechanism of antibacterial action for both isomers is the disruption of the bacterial membrane, which causes bacterial lysis and the leakage of intracellular contents, ultimately leading to cell death. Additional proposed mechanisms include the inhibition of efflux pumps, the prevention and disruption of biofilm formation, the inhibition of bacterial motility, and the inhibition of membrane ATPases [30]. In vivo and in vitro studies by de Souza et al. [31] demonstrated that carvacrol regimens exhibit potent antimicrobial activity against KPC-producing *Klebsiella pneumoniae*, highlighting its potential as a promising candidate for developing alternative therapies. Bnyan et al. [32] investigated the antibacterial activity of carvacrol against nine pathogenic bacteria, including *S. aureus*, *S. epidermidis*, *S. pneumoniae*, *E. coli*, *K. pneumoniae*, *Proteus mirabilis*, *Enterobacter* spp., *Serratia* spp., and *P. aeruginosa*. While carvacrol demonstrated significant growth inhibition across all tested isolates except *P. aeruginosa*, its efficacy was comparable with the standard antibiotic, ciprofloxacin. The study revealed that carvacrol’s mechanism of action is similar to other phenolic compounds, which involves membrane damage, leading to increased membrane permeability and cell wall disruption in both Gram-positive and Gram-negative bacteria. Recent studies have shown that carvacrol can amplify the inhibitory effects of certain conventional drugs, including tetracycline, erythromycin, and fluconazole [33].

Sharifi-Rad et al. [34] reported carvacrol, its impact on human health, and its antimicrobial activity compared to other volatile compounds in EOs. This heightened activity is attributed to a free hydroxy group, its hydrophobic properties, and its phenol structure. However, Miladi et al. [35] recently demonstrated that thymol and carvacrol exhibited strong antibacterial activity against *Vibrio* strains, with thymol showing greater inhibitory effects (MIC: 32–64 mg/mL) against most tested bacteria. When combined with tetracycline or benzalkonium chloride, both compounds significantly reduced MIC values, with thymol achieving a 2- to 8-fold reduction. Additionally, thymol and carvacrol inhibited ethidium bromide efflux in a concentration-dependent manner, highlighting their potential as efflux pump inhibitors in foodborne pathogens. Carvacrol and thymol are effective in inhibiting the growth of both Gram-positive and Gram-negative bacteria. They also exhibit antifungal and antibiofilm properties, making them promising alternative antimicrobial agents against antibiotic-resistant pathogenic bacteria. While their potential for medical applications is acknowledged, further research is necessary to evaluate their toxicity and potential side effects [36].

#### 2.1.2. Menthol

Menthol, also referred to as mint camphor, is a cyclic monoterpene alcohol and a major component of the EO from *Mentha* (peppermint) species (see Table 1). Menthol can interfere with quorum sensing, thereby reducing bacterial pathogenicity. An assessment of menthol’s ability to interfere with quorum sensing systems in various Gram-negative pathogens, which use diverse acyl-homoserine lactone (AHL) molecules, showed that it reduced the AHL-dependent production of violacein, virulence factors, and biofilm formation, indicating broad-spectrum anti-quorum sensing activity. Using two *E. coli* biosensors, pEAL08-2 and MG4/pKDT17, menthol inhibited both the las and pqs quorum sensing systems. Additionally, in vivo studies with menthol on the nematode model of Caenorhabditis elegans demonstrated significantly enhanced nematode survival [37]. Mahmoudi et al. [38] reported that the EO of *Mentha longifolia* and its main component, menthol, exhibited an inhibitory effect on efflux pumps, resulting in a fourfold or greater increase in the susceptibility of *A. baumannii* strains. Combining ciprofloxacin and imipenem with the EO of *Mentha longifolia* and menthol significantly reduced MICs (by 4- to 32-fold) in 25 out of 70 *A. baumannii* isolates (35.71%). Specifically, when combined with ciprofloxacin, MICs decreased fourfold, and with imipenem, they decreased eightfold. Additionally, menthol, when used with imipenem, reduced resistance to imipenem by up to sixteenfold in 90% (63/70) of the isolates.

**Table 1:** Extraction of the selected monoterpenoids and phenylpropanoids from various plant species.

Chemotypes	Plant Species (Family)	Extraction	Isolation Methods	Parts Used	Percentage (%)	References
Thymol	*Coleus amboinicus* L. (Lamiaceae)	Ultrasonic-assisted extraction		Leaves	5.51	[39]
	*Thymus vulgaris* L. (Lamiaceae)	Hydrodistillation and solvent extraction (Hex-EtOAc (3:1))	Column chromatography and HPLC	Leaves and aerial	26.18	[16,40,41]
	*Trachyspermum ammi* L. (Apiaceae)	Microwave-assisted extraction	HPLC and GC-FID/GC-MS	Seeds	59.92–99	[42,43]
	*Satureja Montana* L. (Lamiaceae)	Hydrodistillation and solvent-free microwave extraction	GC-MS	Leaves and stem	22.1	[44]
	*Monarda didyma* L. (Lamiaceae)	Hydrodistillation	GC-FID and GC-MS	Flowering aerial	25.633	[45]
	*Lippia sidoides* L. Cham. (Verbenaceae)	Hydrodistillation	GC-MS	Leaves	76.6	[46]
	*Satureja thymbra* L.(Lamiaceae)	Hydrodistillation	GC-MS	Aerial	29.6	[47]
	*Thymus capitatus* L. (Lamiaceae)	Hydrodistillation	GC-MS	Aerial and leaves	0.51 and 51.22	[48,49]
	*Thymus citriodorus* L. (Lamiaceae)	Hydrodistillation and steam distillation	GC-MS	Leaves	53.46 and 14.43	[50,51]
	*Origanum vulgare* L. (Lamiaceae)	Hydrodistillation and supercritical CO_2_	GC-MS	Aerial, leaves, and flowers	27–65	[52,53,54,55]
	*Thymus zygis* (Lamiaceae)	Hydrodistillation	GC-MS	Leaves	40.67 and 20.69	[56,57]
Carvacrol	*Origanum vulgare* L. (Lamiaceae)	Hydrodistillation and supercritical CO_2_	GC-MS	Aerial, leaves, and flowers	41.79–89.89	[52,53,54,55]
	*Origanum majorana* L. (Lamiaceae)	Hydrodistillation and supercritical CO_2_	GC-MS	Leaves	1.8	[58]
	*Rosmarinus officinalis* L. (Lamiaceae)	Steam distillation	GC-MS	Leaves	66.9	[59,60]
	*Thymus vulgaris* L. (Lamiaceae)	Steam distillation	GC-MS	Leaves	45.5	[61]
	*Thymbra capitata* L. (Lamiaceae)	Hydrodistillation	GC-MS	Seeds and aerial	88.89	[62,63]
	*Satureja thymbra* L. (Lamiaceae)	Hydrodistillation	GC-MS	Aerial	65.2	[64]
	*Satureja montana* L. (Lamiaceae)	Steam distillation and hydrodistillation	GC-FID, MSD, and GC-MS	Leaves and flowering shoots	28.1–87.0	[65,66,67,68]
	*Plectranthus amboinicus* L. (Lamiaceae)	Hydrodistillation and solvent extraction (Hex)	GC-MS and MSD	Aerial and leaves	47.00–88.61	[69,70,71,72,73]
	*Monarda fistulosa* L. (Lamiaceae)	Solvent extraction (MeOH) and steam distillation		Seedlings and aerial	70.24 and 54.83	[74,75]
	*Satureja hortensis* (Lamiaceae)	Hydrodistillation and solvent extraction (MeOH:H_2_O(1:1))	GC-MS	Whole plant and aerial	48.51–50.7	[76,77]
Eugenol	*Syzygium aromaticum* (Myrtaceae)	Hydrodistillation and solvent extraction (Hex, EtOAc)	GC-MS	Cloves	71.09–88.90	[78,79,80]
	*Pimenta racemosa* (Myrtaceae)	Steam distillation and hydrodistillation	GC-MS	Leaves and aerial	34.85 and 87.55	[81,82]
	*Cananga odorata* (Lam.) Hook. F. and Thomson (Annonaceae)	Hydrodistillation	GC-MS	Leaves	8.86	[83]
	*Myrtus communis* L. (Myrtaceae)	Hydrodistillation	GC-MS, GC-FID	Leaves, flowers, and stems	10.11	[84]
	*Myristica fragrans* (Myristicaceae)	CO_2_ supercritical fluid extraction, microwave-assisted hydrodistillation, and steam distillation		Leaves and mace	16.6 and 0.4	[85]
	*Pimenta dioica* (Myrtaceae)	Hydrodistillation	GC-MS	Leaves	65.82	[86]
	*Laurus nobilis* L. (Lauraceae)	Hydrodistillation	GC-MS	Leaves	63.57	[87]
	*Ocimum basilicum* L. (Lamiaceae)	Solvent extraction (hexane/EtOH) and hydrodistillation	GC-MS, GC-FID	Leaves and aerial	25.42–63.1	[88,89,90]
	*Cinnamomum verum* J. Presl (Lauraceae)	Hydrodistillation and soxhlet extraction	GC-MS,	Leaves	93.6	[91]
	*Eugenia caryophyllata* L. (Myrtaceae)	Soxhlet extraction	GC-MS	Clove	70.00–95.00	[92]
Menthol	*Mentha piperita* L. (Lamiaceae)	Hydrodistillation	GC-FID, and GC/MS.	Stem and leaves	61.5	[93]
	*Mentha spicata* L. (Lamiaceae)	Hydrodistillation	GC-MS	Leaves	3.0–14.5	[94]
	*Mentha arvensis* (Lamiaceae)	Hydrodistillation	GC-MS	Leaves	40–45 and 82.3	[95,96]
	*Mentha longifolia* L. (Lamiaceae)	Hydrodistillation	GC-MS	Whole plant	19.4–32.5	[97]
	*Mentha citrate* L. (Lamiaceae)	Hydrodistillation	GC-MS	Leaves	15.4	[98]
	*Cinnamomum verum* L. (Lauraceae)	Hydrodistillation	GC-MS	Bark	60–75	[99,100]
	Cinnamomum burmannii L. (Lauraceae)	Steam distillation	GC-MS	Bark	42.57	[101]
	*Cinnamomum loureiroi* L. (Lauraceae)	Hydrodistillation	GC-FID	Twigs	57.25	[102]
	*Cinnamomum tamala* L. (Lauraceae)	Hydrodistillation and ultrasonic extraction	GC–MS	Leaves	44.49	[103]

### 2.2. Phenylpropanoids

Phenylpropanoids, on the other hand, are a group of organic compounds characterized by a phenyl ring attached to a three-carbon side chain [104]. This class includes compounds such as eugenol and cinnamaldehyde, which are well-known for their antimicrobial properties [105]. The antibacterial activity of phenylpropanoids is attributed to their ability to compromise the integrity of bacterial cell membranes, induce oxidative stress, and modulate bacterial signaling pathways [106]. These mechanisms, along with their relatively low toxicity to humans, make phenylpropanoids promising candidates for developing new antibacterial agents.

#### 2.2.1. Cinnamaldehyde

Cinnamaldehyde shows great potential as a drug for treating drug-resistant *Aeromonas hydrophila* infections. Its bacteriostatic action primarily involves disrupting the integrity of the cell structure, interfering with DNA synthesis and protein metabolism, and impairing overall cellular metabolism [107]. Cinnamaldehyde inhibits the growth of both Gram-positive and Gram-negative bacteria in their planktonic form. Moreover, it also prevents the formation of biofilms, which are closely associated with infections [108]. It has been suggested that the aldehyde functional group in cinnamaldehyde contributes to its antibiofilm activity [109]. Substitutions on the phenyl ring (e.g., halogens or alkyl groups) can improve activity due to increased hydrophobicity [110]. Albano et al.’s study [111] confirmed that cinnamaldehyde effectively reduces the planktonic growth of *Staphylococcus epidermidis*, inhibits biofilm formation, and eradicates pre-formed biofilms. The investigation into cinnamaldehyde’s mechanism of action showed that it affects cell membrane permeability, with confocal laser scanning microscopy images highlighting its role in detaching and killing existing biofilms. A study by Chen et al. [112] revealed that higher concentrations of cinnamaldehyde caused significant morphological changes and disrupted the cell wall and membrane of methicillin-resistant *Staphylococcus aureus* (MRSA). Cinnamaldehyde also compromised membrane integrity, increased outer membrane permeability in a concentration-dependent manner, and induced the release of β-galactosidase, extracellular DNA, and LDH while elevating cellular ROS levels. RT-qPCR analysis showed that cinnamaldehyde at 1× MIC significantly upregulated genes related to fatty acid biosynthesis in MRSA cell membranes, while Western blotting results indicated that cinnamaldehyde suppressed protein expression, highlighting its antibacterial effects. These findings underscore the potent inhibitory action of cinnamaldehyde against MRSA, supporting its potential use as an antibacterial agent, particularly in treating bovine mastitis. Yossa et al. [113] studied the antimicrobial activity of cinnamaldehyde and Sporan, alone or with acetic acid, and tested them against *E. coli* O157:H7 and *Salmonella* in Luria–Bertani broth at 37 °C. Cinnamaldehyde (800 ppm) eliminated both pathogens within one hour, while Sporan (1000 ppm) reduced *Salmonella* and *E. coli* O157:H7 populations by 1.83 and 3.02 log cfu/mL within 2 and 4 h, respectively. Scanning and transmission electron microscopy revealed structural damage and cellular leakage, with cinnamaldehyde showing superior efficacy, while Sporan’s effectiveness depended on concentration, exposure time, and pathogen type. Shu et al. [114] demonstrated that high and low doses of cinnamon EO and cinnamaldehyde significantly improved motility and lifespan and reduced *Pseudomonas aeruginosa* accumulation in *C. elegans* infected with the bacteria. The mechanisms varied, with low doses enhancing the transcription of antimicrobial peptides, and were potentially regulated by the PMK-1-mediated p38 signaling pathway. In vitro antibacterial tests and antioxidant assays further confirmed the potential of cinnamon EO and cinnamaldehyde as effective antibacterial agents. Another study revealed that cinnamon bark oil and cinnamaldehyde exhibited strong bactericidal activity against multidrug-resistant *P. aeruginosa* isolates. Additionally, they demonstrated the promising potential of combining colistin, a drug commonly used to treat Gram-negative bacterial infections, with cinnamon bark oil and cinnamaldehyde [115].

#### 2.2.2. Eugenol

Eugenol demonstrates strong antibacterial activity against *Vibrio vulnificus*, with an MIC of 0.2 mg/mL. Eugenol inhibits *V. vulnificus* growth by compromising cell membrane integrity through oxidative stress, resulting in the leakage of intracellular components. Additionally, eugenol is effective in removing *V. vulnificus* biofilms and successfully prevented contamination in oysters without significantly affecting sensory quality at a concentration of 0.10%. These findings offer insights into eugenol’s mechanism of action against *V. vulnificus* and suggest the potential for natural preservatives as inhibitors of foodborne pathogens [116]. The eugenol extracted from *Syzygium aromaticum* exhibits strong antibacterial and antibiofilm properties against antibiotic-resistant *Helicobacter pylori*, in addition to demonstrating anti-inflammatory activity [117]. A study by Bai et al. [118] suggested that eugenol could be a promising antibacterial agent to address food safety concerns related to *Shigella flexneri.* The research confirmed that eugenol exhibits antibacterial properties against *S. flexneri* by reducing the enzyme activity of superoxide dismutase (SOD), which leads to increased intracellular reactive oxygen species (ROS) levels and oxidative damage to the cell membrane. This damage disrupts the permeability and integrity of the cell membrane, causing leakage of intracellular ATP and a change in membrane potential. Field emission scanning electron microscopy (FESEM) analysis further verified the cell membrane disruption, which ultimately resulted in the death of *Sh. flexneri* cells. Additionally, eugenol was shown to effectively inactivate *Sh. Flexneri* in lysogeny broth (LB), minced pork, and vegetable juice. Ashrafudoulla et al. [119] propose that eugenol could enhance seafood quality by mitigating contamination from *Vibrio parahaemolyticus*. Their research showed that eugenol effectively combats antibiotic-resistant *V. parahaemolyticus* strains. The compound significantly disrupted biofilm formation by interfering with cell-to-cell connections, detaching established biofilms, and reducing the number of viable bacterial cells within the biofilms. Additionally, eugenol increased the leakage of nucleic acids and proteins from *V. parahaemolyticus*, decreased both the metabolic activity and extracellular polymeric substance (EPS) production in biofilms, and potentially reduced the hydrophobicity, motility, and virulence of these isolates. Su et al. [120] confirmed that eugenol holds potential as an antibacterial agent to prevent *Shigella sonnei* contamination in lettuce juice. Their research demonstrated that eugenol exhibits antibacterial properties against *S. sonnei*. They found that eugenol increased intracellular ROS levels in *S. sonnei*, leading to oxidative damage to the cell membrane. Additionally, eugenol caused a reduction in intracellular adenosine triphosphate (ATP) concentration, hyperpolarization of the cell membrane, decreased membrane integrity, and changes in cell morphology.

## 3. Extraction and Isolation Methods

**Figure 2:**
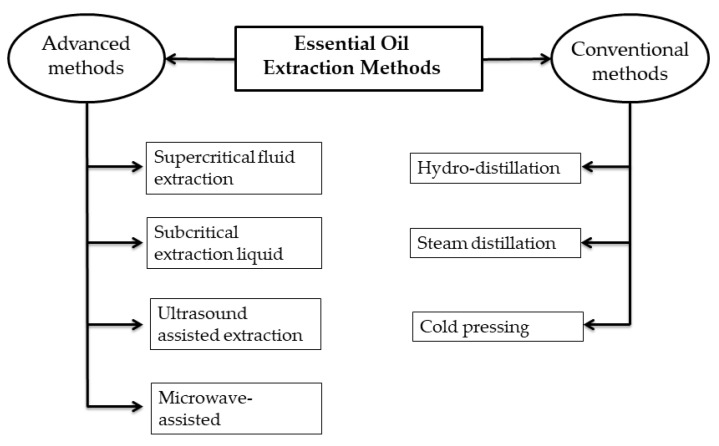
Classification of EO extraction methods.

According to the *European Pharmacopeia* (seventh edition) [121], EOs are “aromatic products consisting of a mixture of compounds extracted from plant raw material, either through dry distillation, steam distillation, or by an appropriate mechanical technique without heating”. Herbal and medicinal plants hold significant importance since a large portion of the population relies on their products, such as EOs. These plant-derived products are utilized in various industries, including medicine, food, and cosmetics [122]. A variety of extraction techniques are employed to obtain EOs from different parts of plant species, including flowers, buds, barks, peels, leaves, and seeds [123]. The techniques used to extract EOs from these plants include steam distillation, solvent-assisted extraction, hydrodistillation, ultrasonic-assisted extraction, supercritical fluid extraction, and solvent-free microwave extraction. Figure 2 provides an overview of the classification of conventional and advanced oil extraction techniques. The choice of an appropriate extraction method for extracting EOs depends on the plant species. Each plant species or plant part has a recommended standard distillation method, such as hydrodistillation, steam distillation, or dry distillation [124]. It is essential to emphasize that the composition of EO is significantly affected by the plant variety, the location where it is grown (including climate and environmental conditions, any stress endured, etc.), and the type of nutrition and fertilizers applied [125].

Conventional methods are less advantageous because EOs are thermolabile, meaning high temperatures can degrade them, and the extract quality is often poor due to the production of chemical artefacts from prolonged extraction times. Hydrodistillation, one of these traditional methods, is straightforward and ancient (used by Avicenna in the 10th century) [124]. With this technique, plant material is directly boiled in water; the setup includes an alembic connected to a condenser (which captures the EO) and a decanter (which collects the evaporated water) [126]. The second technique used for extracting EOs is steam distillation. Although it operates on the same principle as hydrodistillation, the plant material does not come into contact with water. The cold pressing method involves the mechanical pressing of plant parts to release natural oils without applying heat, thereby preserving the oils’ purity and delicate volatile compounds [127,128].

Advanced methods, in contrast to traditional techniques, offer numerous advantages, such as reduced solvent usage and shorter extraction times. One of the most common advanced methods is supercritical fluid extraction. This method typically uses CO_2_ as the solvent, which is compressed and heated to achieve a supercritical state. As it passes through the plant material, the supercritical CO_2_ absorbs volatile components and extracts them. Finally, during the depressurization phase, the oil is separated [129]. Subcritical extraction liquids, such as water and CO_2_, can be used in their subcritical state, allowing extraction at lower temperatures. In ultrasound-assisted extraction, the plant material is immersed in water or selected solvents. This method promotes the release of EOs from the plant due to the mechanical vibration of the cell walls and membranes [130]. Microwave-assisted extraction is one of the most effective and promising methods for extracting EOs. Several variants can be used, including solvent-free microwave extraction (SFME), Microwave Hydro-diffusion and Gravity (MHG), microwave steam distillation (MSD), and microwave steam diffusion (MSDf). When compared with other extraction methods for *Lavandula* EOs proposed by Périno-Issartier et al., MSDf was found to be more efficient in terms of extraction time and produced a higher-quality extract [131].

As shown in Table 1, ongoing research by scientists has identified numerous sources of EOs and their components from various plant species, employing diverse extraction and isolation methods. Since many plant species contain relatively high levels of these components with potential pharmacological properties, researchers often prioritize extraction or isolation over synthesizing these molecules. Since EO components are volatile and non-polar, the most commonly used method for their analysis is GC-MS. GC-FID is utilized to determine the percentage composition of EO components.

## 4. Derivatives of the Selected Monoterpenoids and Phenylpropanoids and Their Impact

The structural modification of natural products has proven to be an effective method for enhancing their antibacterial efficacy, especially in light of the growing prevalence of antibiotic resistance [132]. By modifying the chemical structure of naturally occurring compounds, researchers aim to improve their potency, broaden their spectrum of activity, and reduce potential side effects [133]. These modifications can involve changes, such as the addition or substitution of functional groups, hybridization with other active molecules [134,135], or synthesizing analogs/derivatives. The goal is to optimize the interaction of these compounds with bacterial targets, thereby increasing their efficacy. In the context of EO components, structural modification has proven particularly promising, offering the potential to transform these naturally occurring substances into more effective antibacterial agents [136,137]. This approach not only overcomes the limitations of existing natural compounds but also contributes to developing novel therapeutics that can address the urgent need for new antibacterial drugs. For example, Pinheiro et al. investigated thymol, carvacrol, eugenol, ortho-eugenol, guaiacol, and several chlorinated and allyl phenyl ether derivatives. These compounds are effective against various bacteria, including *S. aureus* and *P. aeruginosa* [138]. Numerous researchers have explored strategies to alter the molecular structure of the selected EO components to enhance their therapeutic effects. These structural modifications in the present study are widely investigated for their potential to improve antibacterial activity. The following sections provide a detailed literature review on selected monoterpenes and phenylpropanoids, highlighting their successfully enhanced antibacterial properties.

### 4.1. Derivatives of Thymol

**Figure 3:**
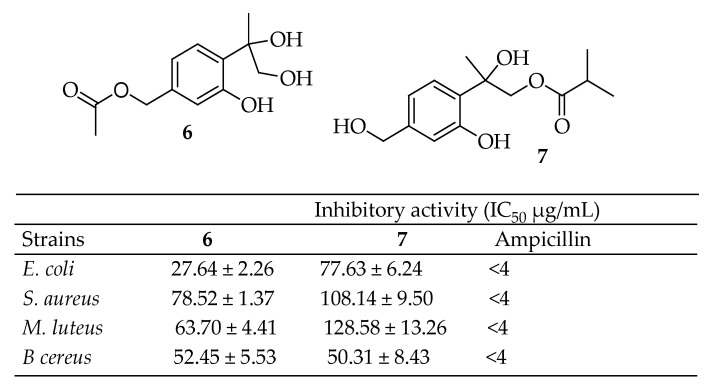
Chemical structures of compounds **6** and **7** and their antibacterial outcomes [139].

Yang et al. [139] identified a new monoterpene compound, 7-acetyl-8,9-dihydroxy thymol (**6**, Figure 3), and a known compound, 7,8-dihydroxy-9-butyryl thymol (**7**, Figure 3), from the dried flower buds of *Lonicera japonica* Thunb. The antibacterial properties of these compounds were tested against *S. aureus*, *E. coli*, *Micrococcus luteus*, and *Bacillus cereus*. Remarkably, both compounds displayed significant antibacterial activity, with IC_50_ values ranging from 27. 64 ± 2.26 to 128. 58 ± 13. 26 μg/mL.

Inspired by the antibacterial characteristics of isatin and thymol, along with the triazole linker’s effectiveness in molecular hybridization, Singh et al. [140] designed, synthesized, and tested a new series of triazole-linked thymol–isatin hybrids for antibacterial activity. The majority of these hybrids demonstrated wide-ranging antibacterial effects, effective against standard human pathogens as well as clinically isolated multidrug-resistant bacteria from the WHO’s “priority pathogen” list and the ESKAPE group.

**Figure 4:**
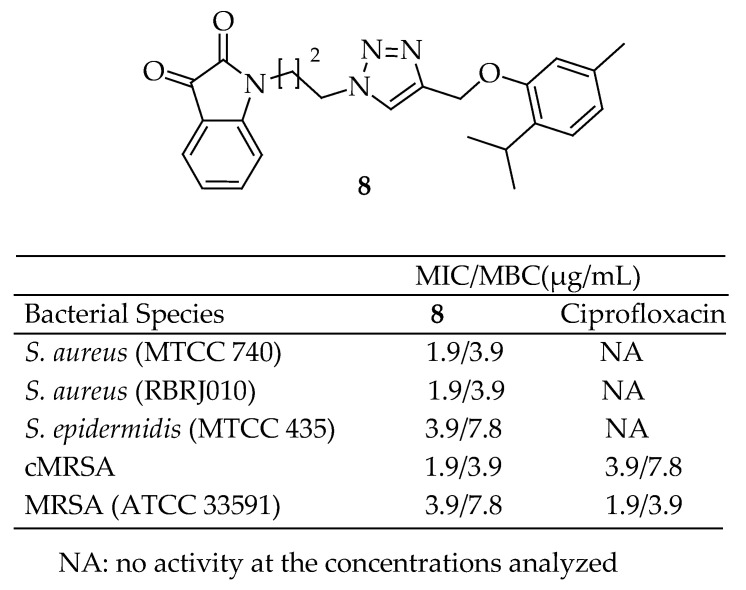
Chemical structure of compound **8** and its antibacterial outcomes [140].

Within the series, hybrid compound **8** in Figure 4 with unsubstituted isatin nucleus stood out for its potent antibacterial activity against MRSA, with a MIC value of 1.9 μM and a minimum bactericidal concentration (MBC) of 3.9 μM, and it also had the ability to inhibit biofilm formation. Compound **8** displayed an inhibitory effect on dehydrosqualene synthase (CrtM) in MRSA, leading to a reduction in the production of staphyloxanthin, a key virulence factor. These findings were further validated through molecular docking and simulation studies. Moreover, **8** proved to be non-toxic and exhibited significant in vivo antibacterial efficacy, with a 90% survival rate at a dose of 10 mg/kg in a larva-based (Galleria mellonella) model of systemic infections. The compound also showed a modulated immune response, indicating its potential to treat MRSA infections effectively. According to structure-activity relationship (SAR) analysis, substitutions at the fifth position of the isatin moiety significantly influence antibacterial activity. Electronegative halogen substitutions at this position, including fluorine, chlorine, bromine, and iodine, appear to be detrimental to the antibacterial efficacy of the structure.

Additionally, altering the chain length between the isatin and triazole nucleus from three carbons decreased the antibacterial efficacy. For electropositive substitutions, a slight increase in electronegativity led to reduced antibacterial efficacy. Conversely, with electronegative halogen substitutions, the antibacterial activity increases with greater electronegativity, although the difference is not substantial.

**Figure 5:**
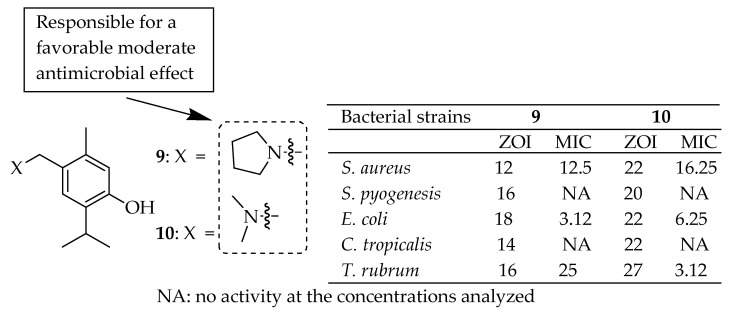
Chemical structures of compounds (**9** and **10**) and their antibacterial outcomes [141].

Bishoyi et al. [141] synthesized thymol Mannich-base derivatives and evaluated their antibacterial activity against uropathogenic bacteria, *S. aureus*, *S. pyogenes*, and *E. coli*. Among these, compound **9** (depicted in Figure 5) exhibited significant antibacterial activity. Specifically, it showed the notable inhibition of *E. coli* and *S. aureus* with MICs of 3.12 μg/mL and 12.5 μg/mL, respectively. Another compound, **10** in Figure 5, exhibited effective inhibition against *E. coli* and *S. aureus* with MICs of 6.25 μg/mL. The SARs of these derivatives indicated that phenolic thymol structures with simple aminomethyl substitutions exhibited a favorable moderate antimicrobial effect.

**Figure 6:**
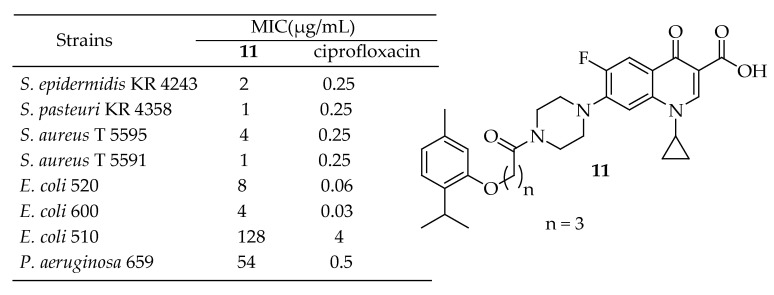
Chemical structure of compound **11** and its antibacterial outcomes [142].

Szostek et al. [142] successfully synthesized sixteen new ciprofloxacin-containing thymol or menthol and evaluated their activity against Gram-negative and Gram-positive bacterial strains, as shown in Figure 6. The compounds exhibited similar potency against *E. coli* 520 and 600, with MIC values ranging from 4 to 64 µg/mL. For Gram-positive hospital microorganisms, all tested derivatives were active, with MIC values between 1 and 16 µg/mL, indicating high antimicrobial potency. Compound **11** (Figure 6) emerged as the leading structure, with MICs of 1 µg/mL against *S. pasteuri* KR 4358 and *S. aureus* T 5591 (clinical strains). Interestingly, no trends were observed in relation to the linkers used (ranging from acetyl to hexyl). The best results were obtained for compound **11** from the thymol group, which contained a butyl linker. This study revealed poor outcomes for disubstituted derivatives, indicating that the 3-oxo-4-carboxylic acid core is the active DNA gyrase-binding site. Structural modifications to this fragment were associated with a decrease in antibacterial efficacy.

**Figure 7:**
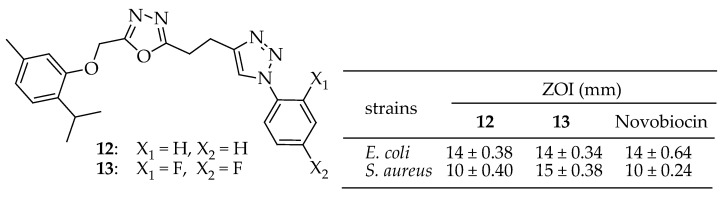
Chemical structures of compounds (**12** and **13**) and their antibacterial outcomes [143].

Almalki et al. [143] synthesized and evaluated 1,2,3-triazole-containing thymol-1,3,4-oxadiazole derivatives with antimicrobial activities against *E. coli* and *S. aureus*. Derivatives **12** and **13** (Figure 7) exhibited the highest potency against *E. coli*, each showing zones of inhibition of 14 mm. Additionally, compound **13** displayed the greatest activity against *S. aureus*, with a zone of inhibition (ZOI) of 15 mm. The synthesized 1,2,3-triazole-linked thymol-1,3,4-oxadiazole conjugates exhibit antimicrobial properties. These derivatives caused significant inhibition of the thymidylate synthase enzyme.

**Figure 8:**
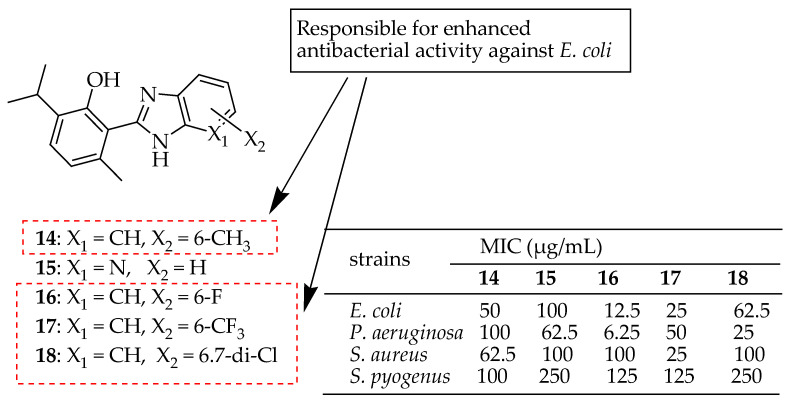
Chemical structures of compounds (**14**–**18**) and their antibacterial outcomes [144].

Bhoi et al. [144] developed a series of benzimidazole derivatives derived from the naturally occurring phenolic monoterpenoid thymol, utilizing a green synthesis approach. These derivatives were evaluated for their antibacterial activity against *Escherichia coli*, *P. aeruginosa*, *S. aureus*, and *S. pyogenes*. Among these derivatives, compounds **14**–**18** in Figure 8 demonstrated significant antibacterial activity against the *E. coli* strain. Compounds **15**–**18** showed potent antibacterial activity against the *P. aeruginosa* strain. Compounds **14** and **17** exhibited considerable antibacterial activity against the *S. aureus* strain. The in silico analysis of ADME characteristics for the synthesized compounds suggested they would make excellent drugs with high oral bioavailability. According to the SAR analysis, the alkyl, chloro, and fluoroalkyl groups (in compounds **14** and **16**–**18**) were responsible for enhanced antibacterial activity against *E. coli*. The benzimidazole core, when featuring alkyl and fluoroalkyl groups (**14** and **17**) at the sixth position, demonstrated increased antibacterial activity against *S. aureus.*

**Figure 9:**
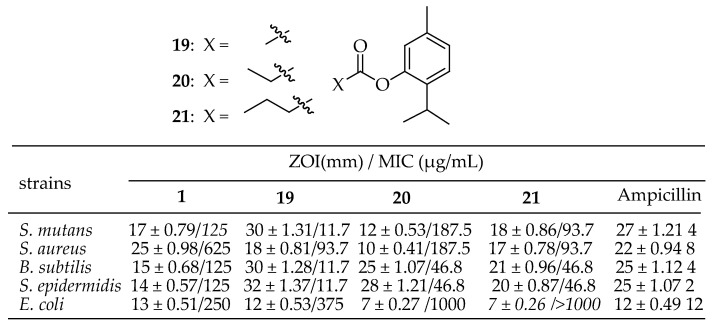
Chemical structures of compounds (**19**–**21**) and their antibacterial outcomes [145].

Mathela et al. [145] synthesized and characterized fourteen esters of thymol and carvacrol using spectral data. The antibacterial activity of thymol, carvacrol, and their esters against four Gram-positive bacteria (*S. mutans* MTCC 890, *S. aureus* MTCC 96, *B. subtilis* MTCC 121, and *S. epidermidis* MTCC 435) and one Gram-negative bacterium (*E. coli* MTCC 723) was studied. The thymol ester derivatives **19**–**21** in Figure 9 showed increased activity against *S. mutans*, *B. subtilis*, and *S. epidermidis* compared to thymol, while the carvacrol derivatives exhibited significantly less activity than carvacrol.

**Figure 10:**
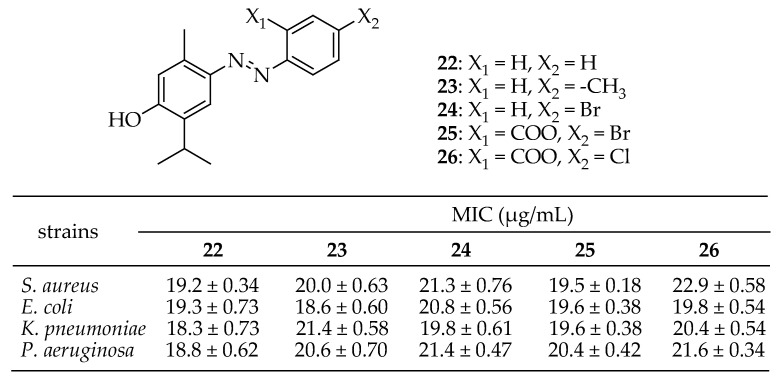
Chemical structures of compounds (**22**–**26**) and their antibacterial outcomes [146].

Swain et al. [146] synthesized twelve derivatives of aryl-azo-thymol and evaluated the antimicrobial properties of these compounds using the agar well diffusion method against four bacterial strains: *S aureus*, *E. coli*, *K pneumonia*, and *P. aeruginosa*. Additionally, drug-likeness properties, as well as their toxic nature and LD_50_ values, were studied through bioinformatic tools like PASS prediction, molecular docking, and the Lipinski rule of five. Out of the 12 derivatives, compounds **22**–**26** in Figure 10 showed notable antibacterial activities with MIC values ranging from 40 to 80 μg/mL. The docking scores of these derivatives ranged from −8.27 to −11.44 kcal/mol against β-lactamase species of the selected bacterial strains. Based on both in vitro and in silico studies, these thymol derivatives were found to be effective against MRSA and ESBL-producing bacteria.

**Figure 11:**
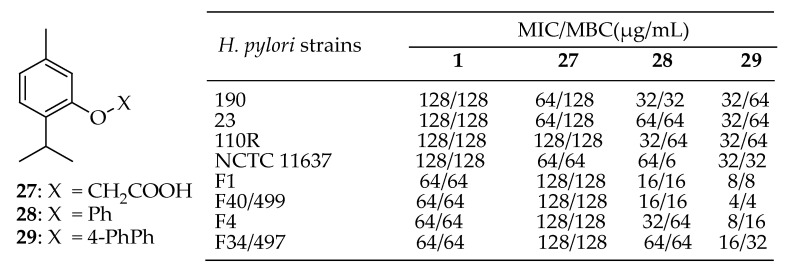
Chemical structures of compounds (**27**–**29**) and their antibacterial outcomes [147].

Sisto et al. [147] investigated a molecular library based on thymol with significant structural diversity to find new dual-action agents that can both inhibit the growth of various strains of *H. pylori* and exhibit toxicity towards human gastric adenocarcinoma. Their examination of the structure-activity relationships within this library revealed that certain structural modifications to the OH group of thymol resulted in a broader range of growth inhibition across all isolates. The preferred modifications were benzyl groups over alkyl chains, with most being effective anti-*H. pylori* when functional groups were present at the para position of the benzyl moiety, such as 4-CN and 4-Ph. These modifications led to MIC values as low as 4 μg/mL for all *H. pylori* strains tested. Three derivatives from this library, compounds **27**–**29** in Figure 11, have emerged as promising lead candidates for alternative therapies against *H. pylori* infections, potentially reducing the risk of severe gastric diseases.

**Figure 12:**
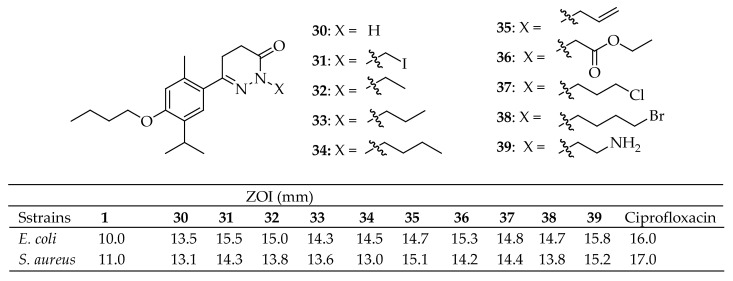
Chemical structures of compounds (**30**–**39**) and their antibacterial outcomes [148].

Nagle et al. [148] developed new pyridazinone derivatives containing thymol and assessed their antimicrobial properties in vitro. The antimicrobial activities were measured using the agar well diffusion technique against the bacteria *E. coli* and *S. aureus*. Of all the pyridazinone compounds they synthesized, the compounds labeled **30**–**39** in Figure 12 showed significant antibacterial activity. To further explore the potential of these compounds, virtual screening was conducted through docking studies targeting glucosamine-6-phosphate synthase and *Aspergillus niger* phytase. The results indicated that nearly all the synthesized compounds demonstrated strong binding affinity to these receptors, outperforming the original compound (thymol). The study also demonstrated that incorporating a pyridazinone ring into thymol significantly boosts its antibacterial activity compared to thymol alone.

**Figure 13:**
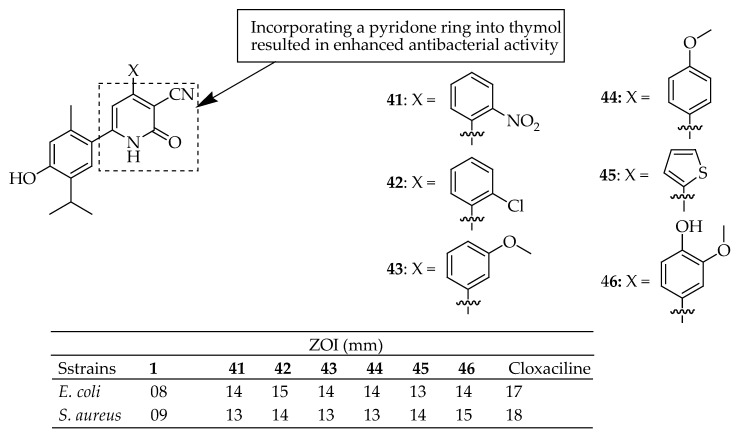
Chemical structures of compounds (**41**–**46**) and their antibacterial outcomes [149].

Nagle et al. [149] synthesized and characterized a range of derivatives from 1,2-dihydro-6-(4-hydroxy-5-isopropyl-2-methylphenyl)-2-oxo-4-arylpyridine-3-carbonitrile. They assessed these compounds for antioxidant and antimicrobial properties through in vitro experiments. Among the new derivatives, compounds **41**–**46** in Figure 13 showed better antibacterial inhibition against *E. coli* and *S. aureus* species. However, all derivatives demonstrated superior antibacterial properties compared to thymol, the parent compound. The investigation also indicates that incorporating a pyridone ring into thymol enhanced antibacterial activity compared to thymol alone.

**Figure 14:**
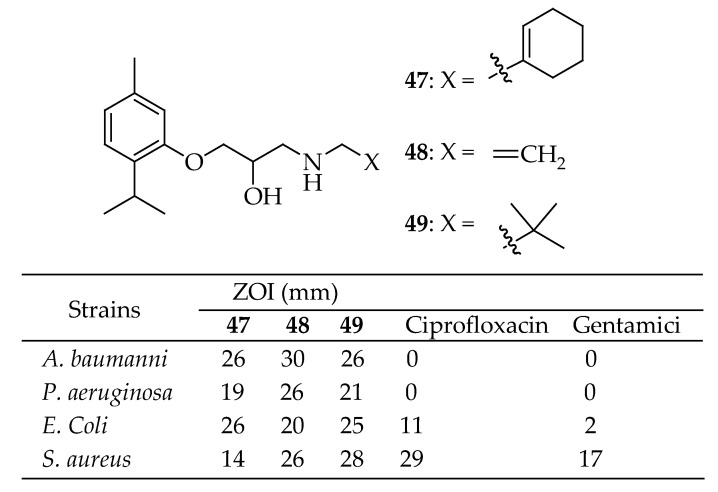
Chemical structures of compounds (**47**–**49**) and their antibacterial outcomes [150].

A series of novel oxypropanolamine-based compounds containing thymol were synthesized and analyzed by Zengin et al. [150]. The agar well diffusion method was used to test their antibacterial properties against strains of *A. baumannii*, *P. aeruginosa*, *E. coli*, and *S. aureus* in an in vitro setting. Compounds **47**–**49** in Figure 14 demonstrated the most potent antibacterial activity among all those tested, with nearly all showing greater efficacy than the reference drugs. These newly synthesized thymol-based oxypropanolamine derivatives exhibited strong inhibitory effects on α-glycosidase, cytosolic carbonic anhydrase I and II isoforms (hCA I and II), and acetylcholinesterase enzymes (AChE), with Ki values ranging from 463.85 to 851.05 µM for α-glycosidase, 1.11 to 17.34 µM for hCA I, 2.97 to 17.83 µM for hCA II, and 13.58 to 31.45 µM for AChE, respectively. Among all the compounds tested, **47**–**49** displayed the most pronounced antibacterial effects, surpassing the antibacterial activity of most reference drugs. These results showed that AChE’s inhibition by the novel thymol-bearing oxypropanolamine derivatives (**47**–**49**) is much better than that of standard drugs.

**Figure 15:**
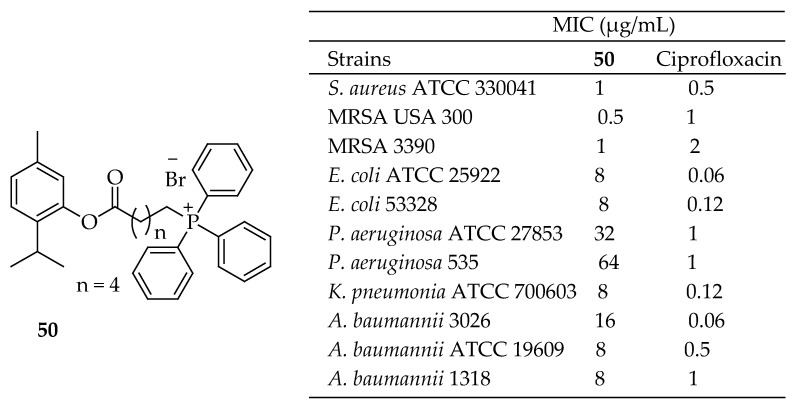
Chemical structure of compound (**50**) and its antibacterial outcomes [151].

A series of thymol triphenylphosphine (TPP) conjugates were designed and synthesized by Tang et al. [151] and evaluated against several Gram-positive bacteria (*S. aureus* ATCC 43300, MRSA USA 300, MRSA 3390) and Gram-negative bacteria (*E. coli* ATCC 25922, *E. coli* 53328, *P. aeruginosa* ATCC 27853, *P. aeruginosa* 535, *K. pneumonia* ATCC 700603, *K. pneumonia* 3026, *A. baumannii* ATCC 19606, and *A. baumannii* 1318). These compounds demonstrated significantly enhanced inhibitory activity against Gram-positive bacteria compared to thymol. Notably, **50** in Figure 15 exhibited a low likelihood of resistance development and showed excellent biocompatibility. Interestingly, at high concentrations, **50** achieved a rapid killing effect on MRSA persisters (99.999%). Fluorescence assays, electron microscopy, molecular dynamics simulations, and bilayer experiments confirmed that **50** conjugates exert potent antimicrobial activity by disrupting bacterial membrane integrity, even in persister cells. Additionally, **50** showed significant efficacy in a mouse model of subcutaneous MRSA infection. It was determined that the antibacterial potency of compounds could be significantly enhanced by conjugating them with the TPP+ group, offering a promising approach for future compound design and modification.

**Figure 16:**
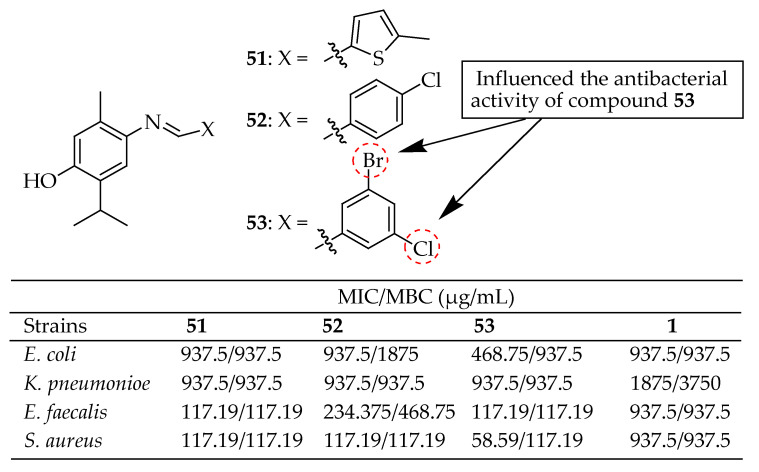
Chemical structures of compounds (**51**–**53**) and their antibacterial outcomes [152].

Thymol hybrid compounds **51**–**53** (shown in Figure 16) were evaluated for antibacterial activity against *E. coli*, *K. pneumoniae*, *E. faecalis*, and *S. aureus.* These compounds exhibited enhanced antibacterial properties, particularly against *E. coli*, with MIC values ranging from 10.66 to 11 μg/mL, comparable to those of their parent compound thymol, as reported by Sahin et al. [152]. Among these, compound **53**, containing two halogens, was identified as the most effective against the mentioned bacterial strain. The presence of halogens in the side chain of the benzylideneamino group influenced the antibacterial activity of compound **53**. Consequently, thymol hybrids represent a promising lead in developing a new generation of antibacterial drugs.

**Figure 17:**
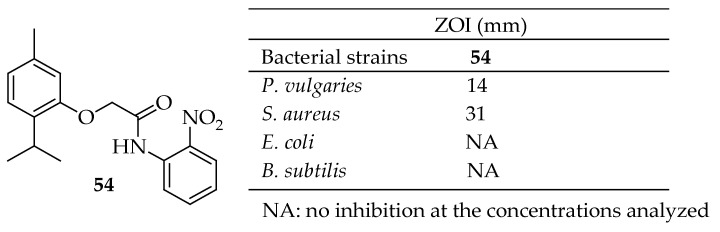
Chemical structure of compound (**54**) and its antibacterial outcomes [153].

Patil et al. [153] developed a range of thymol ether hybrids and tested their efficacy against four different bacterial strains, such as *P. vulgaries*, *S. aureus*, *E. coli*, and *B. subtilis*. Among these, hybrid **54** (depicted in Figure 17) demonstrated significant antibacterial activity, producing a 14 and 31-mm zone of inhibition against *P. valgaries* and *S. aureus*. However, it was ineffective against *E. coli*. Despite the hybrids’ selectivity for certain bacterial strains, incorporating the thymol component enhanced the antibacterial activity.

**Figure 18:**
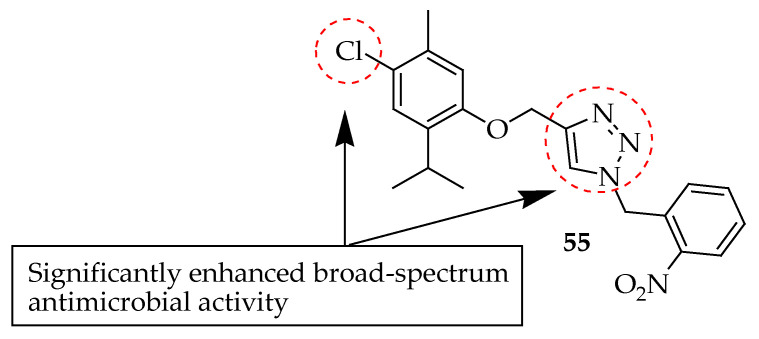
Chemical structure of compound (**55**) and its antibacterial outcomes [154].

Addo et al. [154] synthesized ten 1,2,3-triazole-thymol derivatives from thymol using a click reaction between the O-propargyl terminal alkyne of thymol and chlorothymol with benzyl azide and substituted benzyl azides. All derivatives exhibited significant but varied antibacterial activity. Antibacterial studies of the compound lacking chlorine at the fourth position of the thymol group against *K. pneumoniae* showed that the bacterial strain developed resistance. Therefore, substituting hydrogen with a halogen enhanced the antibacterial efficacy of the compounds. Notably, compound **55** in Figure 18 showed the highest antibacterial activity, with a mean ZOI of 38.7 mm, surpassing the 30.0 mm zone size of ampicillin, the positive control. Additionally, this compound was three times more potent than the parent compound thymol (11.0 mm) against MRSA at a concentration of 100 μg/mL. The antimicrobial activity indicates that the inclusion of a triazole moiety in the thymol nucleus, along with the substitution of chlorine (–Cl) on the thymol nucleus and chlorine (–Cl), fluorine (–F), and nitro (–NO_2_) groups on the aromatic nucleus of thymol, significantly enhanced broad-spectrum antimicrobial activity compared to the parent compound, thymol.

**Figure 19:**
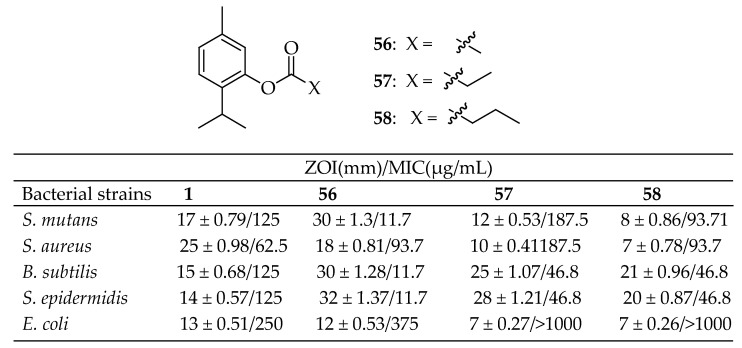
Chemical structures of compounds (**56**–**58**) and their antibacterial outcomes [145].

Mathela et al. [145] conducted a comprehensive study on a series of ester derivatives of carvacrol, evaluating their in vitro antibacterial activity against various pathogens, including *S. epidermidis*, *S. mutans*, *S. aureus*, *B. subtilis*, and *E. coli.* The most significant increase in activity was observed for thymyl ester derivatives **56** and **57** in Figure 19 against Gram-positive bacterial strains. Thymyl acetate **56** and thymyl isobutyrate **57** were more effective than thymol (ZOI = 17 mm; MIC = 125 µg/mL) and all other esters against *S. mutans* (ZOI = 30 mm and 18 mm; MIC = 11.7 and 93.7 µg/mL, respectively), *B. subtilis* (ZOI = 30 mm and 21 mm; MIC = 11.7 and 46.8 µg/mL, respectively), and *S. epidermidis* (ZOI = 32 mm and 20 mm; MIC = 11.7 and 46.8 µg/mL, respectively). Additionally, derivative **58** in Figure 19 exhibited higher activity against *B. subtilis* (ZOI = 25 mm; MIC = 46.8 µg/mL) and *S. epidermidis* (ZOI = 28 mm; MIC = 46.8 µg/mL) compared to thymol.

### 4.2. Derivatives of Carvacrol

Carvacrol has become a prominent agent due to its capability to inhibit bacterial growth, drawing significant interest from researchers. This focus has led to an increasing effort to develop carvacrol derivatives to enhance their antibacterial potency. These derivatives offer a promising avenue to overcome carvacrol’s inherent limitations, enhance its antimicrobial properties, and broaden its application in combating bacterial infections.

**Figure 20:**
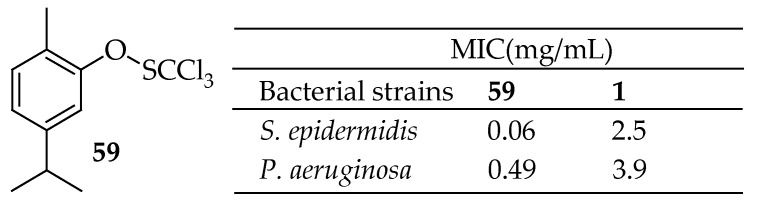
Chemical structure of compound (**59**) and its antibacterial outcomes [155].

Walsh et al. [155] successfully synthesized the sulfenate ester derivative of carvacrol (**59** shown in Figure 20) by replacing the –OH group with a trichloromethylanesulfenate group. These derivatives exhibited enhanced activity against mature biofilms of *S. epidermidis* and *P. aeruginosa*, with MIC values of 0.06 and 0.49 mg/mL, respectively. This advancement highlights the potential of structural modifications to boost antibacterial efficacy significantly, opening new possibilities for improving the therapeutic application of carvacrol derivatives.

**Figure 21:**
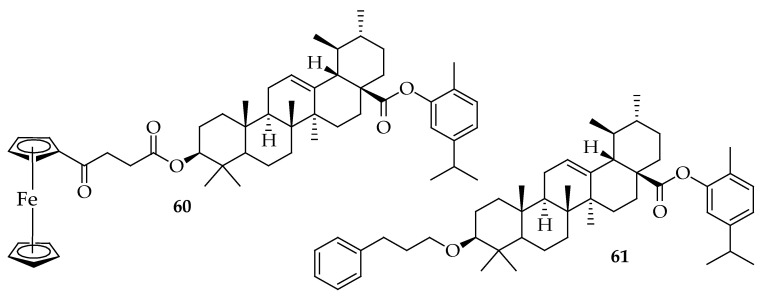
Chemical structures of compounds **60** and **61** and their antibacterial outcomes [156].

We recently synthesized several hybrid molecules and tested their antibacterial effects on specific bacterial pathogens such as *B subtilis* (ATCC19659), *E. faecalis* (ATCC13047), *M. smegmatis* (MC2155), *S. epidermidis*, *S. aureus* (ATCC25923), *Enterobacter cloacae* (ATCC13047), *P. vulgaris* (ATCC6380), *E. coli* (ATCC25922), *Klebsiella oxytoca* (ATCC8724), *Proteus mirabilis* (ATCC7002), *P. aeruginosa* (ATCC27853), and *Pseudomonas aeruginosa* (ATCC 2785) [156]. Out of these hybrids, compounds **60** and **61** (Figure 21) with fused carvacrol showed notable antibacterial activity with MIC values of 15.63 μg/mL against *Proteus vulgaris* and *Proteus mirabilis*.

**Figure 22:**
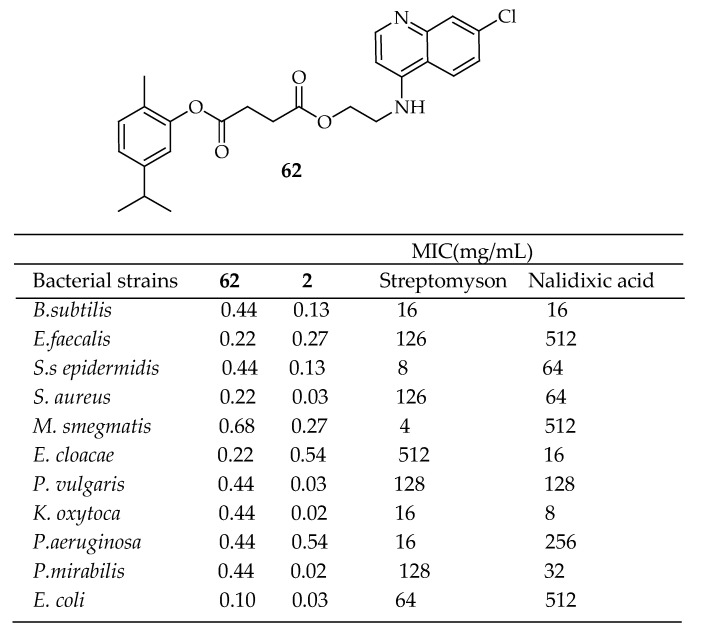
Chemical structure of compound **62** and its antibacterial outcomes [157].

Mbese et al. [157] developed carvacrol ester hybrids (illustrated in Figure 18) and evaluated their effectiveness against various bacterial strains, including *B subtilis* (ATCC19659), *E. faecalis* (ATCC13047), *M. smegmatis* (MC2155), *S. epidermidis*, *S. aureus* (ATCC25923), *Enterobacter cloacae* (ATCC13047), *P. vulgaris* (ATCC6380), *E. coli* (ATCC25922), *Klebsiella oxytoca* (ATCC8724), *Proteus mirabilis* (ATCC7002), *P. aeruginosa* (ATCC27853), and *Pseudomonas aeruginosa* (ATCC 2785). These hybrids exhibited strong antibacterial activity, with MIC values ranging from 1.25 to 3.3 μg/mL. Despite this, they did not show more significant antibacterial effects than carvacrol. Notably, compound **62** in Figure 22 emerged the most potent, with MIC values between 0.10 and 0.68 μg/mL. The 4-aminoquinoline framework played a crucial role in the antibacterial properties of this compound. These conclusions were also supported by the findings of Ranjbar-Karimi Alireza [158].

**Figure 23:**
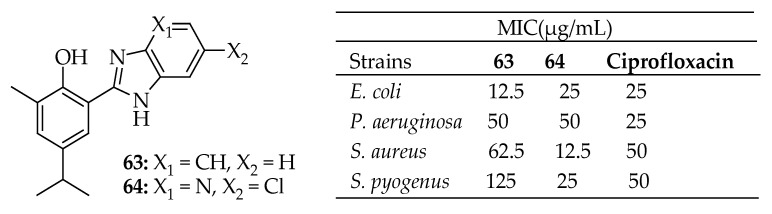
Chemical structures of compounds (**63** and **64**) and their antibacterial outcomes [159].

Bhoi et al. [159] synthesized nine hybrids of benzimidazole and carvacrol and tested them against four bacterial strains: *E. coli*, *S. aureus*, *S. pyogenes*, and *P. aeruginosa*. These hybrids exhibited significant antibacterial activity against all four strains, outperforming some of the control drugs. They demonstrated selectivity towards the bacterial strains with MIC values ranging from 12.5 to 250 µg/mL. Compounds **63** and **64** in Figure 23 demonstrated remarkable antibacterial activity, with **64** being the most potent. It exhibited MIC values of 12.5 µg/mL against *S. aureus*, 25 µg/mL against *E. coli*, 50 µg/mL against *S. pyogenes*, and 25 µg/mL against *P. aeruginosa*. The position and nature of the substituents on the benzimidazole moiety, such as fluoroalkyl and alkyl groups, influenced the antibacterial activity. However, the effect varied depending on the bacterial strain. This study suggests that hybridizing natural products with azoles and modifying their structure can yield potent antibacterial agents capable of tackling resistant bacterial strains.

### 4.3. Menthol Containing Hybrids

**Figure 24:**
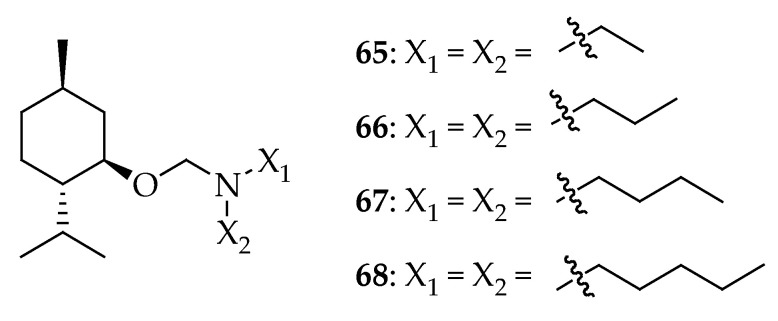
Chemical structures of compounds (**65**–**68**) [160].

Ismailova et al. [160] developed new chiral menthol-containing Mannich bases by condensing L-(–)-menthol with secondary alkyl amines in formaldehyde. These synthesized compounds **65**–**68** in Figure 24 demonstrated high antibacterial activity against microorganisms such as *S. aureus*, *P. aeruginosa*, and *E. coli*, compared to control drugs (ethanol, rivanol, furacilinum, carbolic acid, and chloramine) commonly used in medical practice. The MIC and MBC values of the synthesized compounds were determined, revealing that they were effective against bacteria *S. aureus* at very low concentrations.

**Figure 25:**
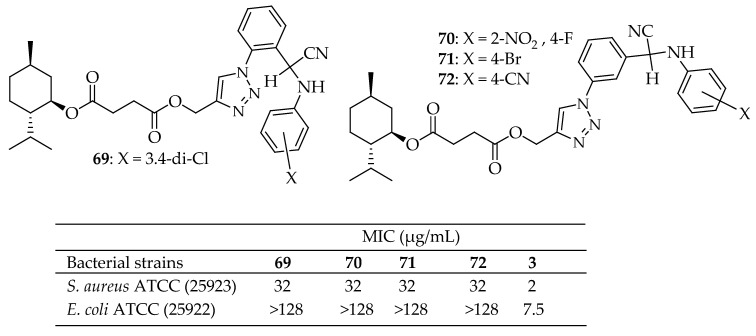
Chemical structures of compounds (**69**–**72**) and their antibacterial outcomes [161].

Karbasi et al. [161] synthesized a library of novel 1,2,3-triazole-tethered menthol derivatives featuring α-aminonitriles through a combination of Huisgen 1,3-dipolar cycloaddition and Strecker reactions. The antibacterial activity of these compounds was evaluated against *S. aureus* and *E. coli*. Most of the synthesized derivatives exhibited more potent activity than menthol, although none matched the efficacy of cefixime. These compounds showed a significant inhibitory effect against *S. aureus*, with MIC values ranging from 32 to 128 μg/mL. Notably, derivatives **69**–**72** displayed the best inhibitory effects with a MIC value of 32 μg/mL (Figure 25).

**Figure 26:**
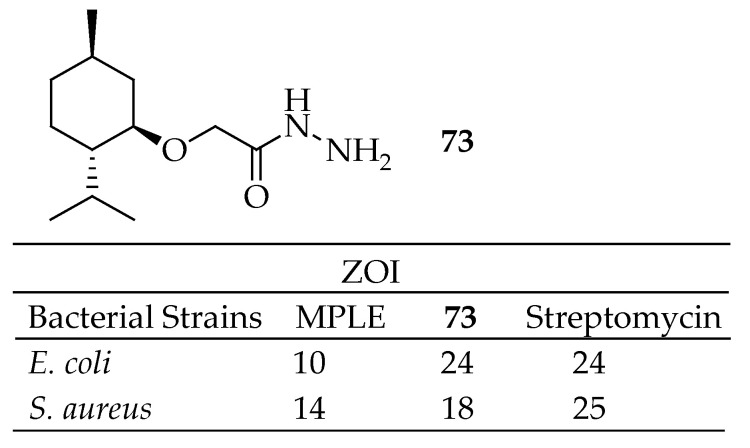
Chemical structure of compound (**73**) and its antibacterial outcomes [162].

Manogaran et al. [162] evaluated the antimicrobial potential of a hydrazide derivative of menthol (**73** in Figure 26) and *Mentha piperita* leaf extract (MPLE) against bacteria that trigger periimplantitis, specifically *E. coli* and *S.aureus*. Their tests revealed that the hydrazide derivative of menthol exhibited significantly higher antimicrobial activity than the *Mentha piperita* leaf extract. Based on these results, they concluded that the hydrazide derivative of menthol has high antimicrobial potential against periimplantitis-triggering bacteria and recommended further evaluation to support its clinical relevance.

**Figure 27:**
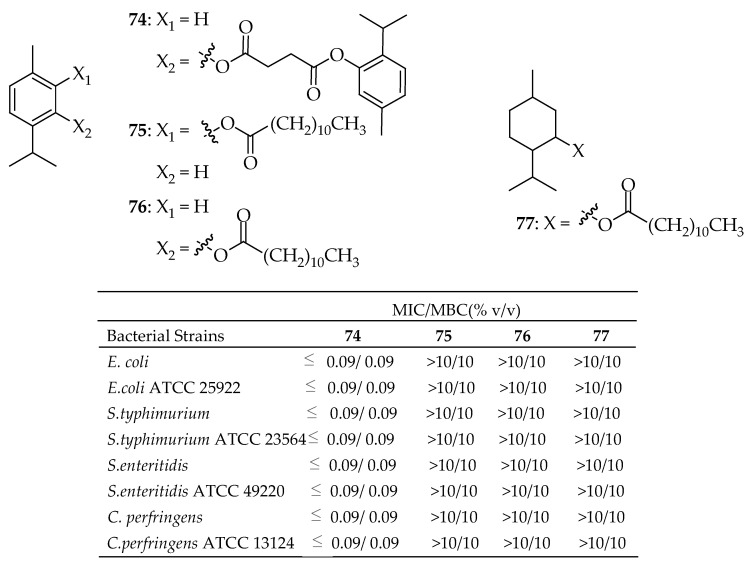
Chemical structures of compounds (**74**–**77**) with their antibacterial outcomes [163].

In a study by Bassanetti et al. [163], the synthesis and evaluation of the antibacterial activity of new thymol, carvacrol, and menthol derivatives were presented. These new compounds were designed to address the limitations of their precursors, such as poor water solubility and volatility, while maintaining a strong antimicrobial profile. The synthesized compounds were tested against pathogens responsible for significant foodborne diseases (see Figure 27), including *Clostridium perfringens*, *Salmonella* Typhimurium, *Salmonella* Enteritidis, and *E. coli*. The compounds demonstrated low MIC and MBC values, good water solubility, and negligible cytotoxicity towards HT-29 human cells, confirming their potential use as EO derivatives in the food industry. Among all the derivatives, thymol succinate **74** demonstrated the highest activity against all tested strains, showing antibacterial properties comparable to the parent compound, with MIC and MBC values of ≤0.09 (% *v*/*v*). In contrast, the mono-esters (compounds **75**, **76**, and **77**) showed limited antimicrobial activity, with MIC values of approximately 10%. Additionally, it is worth highlighting that compound **8** also possesses a superior organoleptic profile.

**Figure 28:**
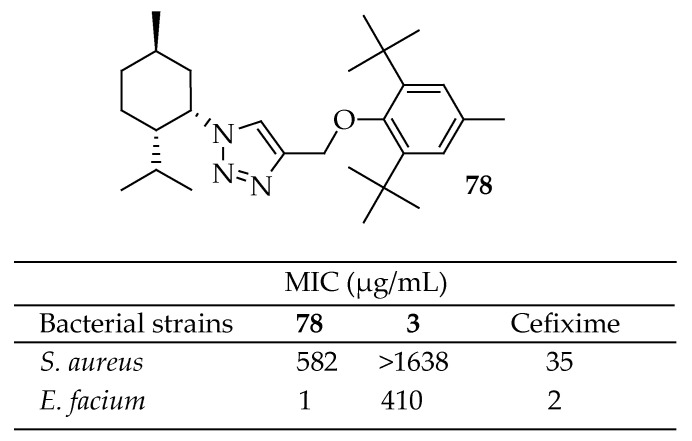
Chemical structure of compound (**78**) and its antibacterial outcomes [164].

Khaligh et al. [164] developed an efficient method for synthesizing a library of 1,4-disubstituted 1,2,3-triazoles based on hydroxybenzaldehydes, phenols, and bile acids, aiming to explore their synergistic potential with menthol. Preliminary studies on menthol and the 1,2,3-triazole analogues provided a promising platform for discovering derivatives with enhanced antibacterial activity. Fifteen derivatives were synthesized via 1,3-dipolar cycloaddition reactions and assessed for their antibacterial activity against *E. faecium* and *S. aureus*. All derivatives exhibited stronger inhibitory activity than the lead compounds against *E. faecium*, and nearly all of them were more potent than cefixime, used as a positive control. Among the derivatives, compound **78** in Figure 28 demonstrated the highest activity. The results indicated that the 1,2,3-triazole ring could effectively enhance the antibacterial activity of lead compounds through various mechanisms. Additionally, the evaluation of the physicochemical properties of the target compounds based on Lipinski’s rule of five demonstrated that almost all compounds had a desirable profile to be considered new lead candidates.

### 4.4. Derivatives of Eugenol

Eugenol is a phenolic compound present in various plants, including clove oil, nutmeg oil, cinnamon extract, and several others. Eugenol was initially extracted from the leaves and buds of clove (*Eugenia caryophyllata*). It possesses various pharmacological properties, including antimicrobial, anti-inflammatory, antioxidant, and antidiabetic properties [165]. Eugenol is classified as “generally recognized as safe (GRAS)” by the FDA and WHO and is permitted for use as a food additive [166,167]. It is extracted through various methods, including steam distillation, solvent extraction, hydrodistillation, microwave-assisted extraction, ultrasound-assisted extraction, and supercritical CO_2_ extraction.

The polyphenol eugenol extracted in clove oils exhibits antibacterial activity against Gram-negative and Gram-positive bacteria. Eugenol disrupts the cell membrane and cell wall of these bacteria, leading to cell lysis and the leakage of intracellular fluids, along with lipid and protein components [168]. Eugenol has attracted significant attention due to its antibacterial properties, and various scientists worldwide have developed eugenol derivatives to enhance its potential. Derivatives of eugenol, including its acetates and methoxy derivatives, have been shown to enhance its antimicrobial efficacy, with some modifications leading to improved potency and reduced toxicity.

**Figure 29:**
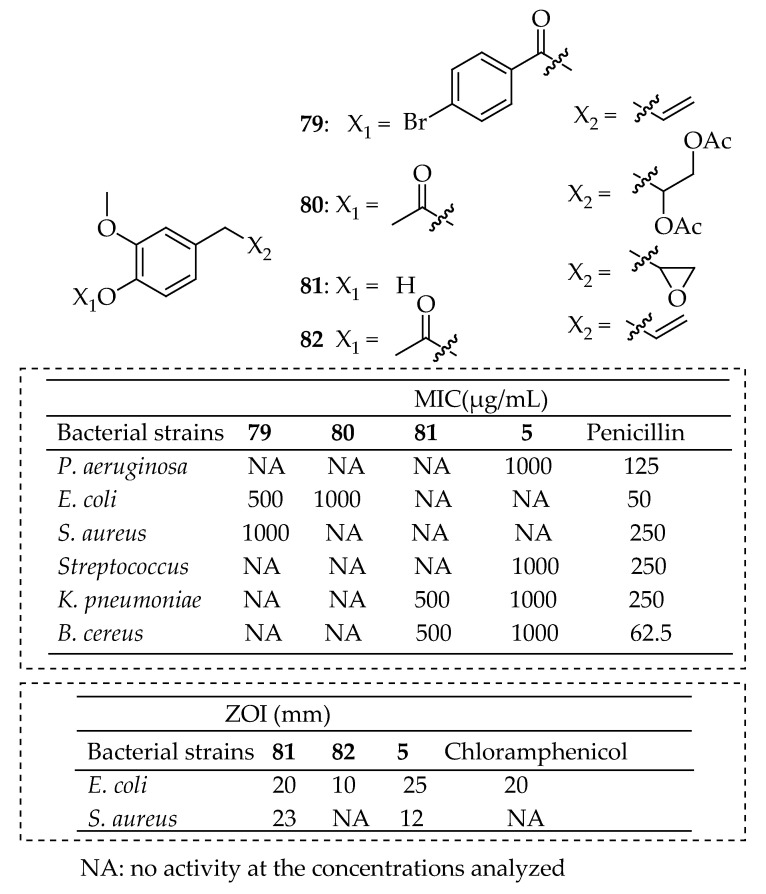
Chemical structures of compounds (**79**–**82**) and their antibacterial outcomes [169,170].

In a recent study, da Silva et al. [169] synthesized eugenol derivatives through the esterification of the hydroxy group (OH) of eugenol with various carboxylic acids, as well as through addition reactions at the double bond of the allyl group. Among these derivatives, compounds **79**–**81** in Figure 29 exhibited promising antibacterial activity, showing a lower MIC of 500 μg/mL compared to eugenol’s 1000 μg/mL. These derivatives were effective against bacterial strains, such as *E. coli* and *S. aureus*, in which eugenol alone showed no activity, thereby broadening the spectrum of antibacterial action. Abdou et al. [170] synthesized two eugenol derivatives, acetyl eugenol (**83**, in Figure 29) and epoxy eugenol (**82**), and evaluated their antibacterial activity against *E. coli* and *S. aureus* using the inhibition zone technique. Eugenol’s effect against *E. coli* was high, with a zone of inhibition of 25 mm. The epoxidation of eugenol enhanced its activity against *S. aureus*. In contrast, acetyl eugenol was inactive against *S. aureus* and showed the lowest activity against *E. coli*. Molecular docking analysis of the three compounds and identifying amino acids in the active site pockets of the target proteins from both bacteria further provided insights into the observed biological activity.

**Figure 30:**
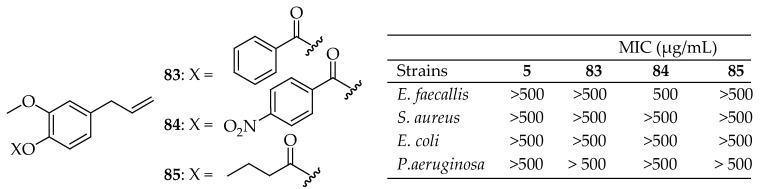
Chemical structures of compounds **83**–**85** and their antibacterial outcomes [171,172].

Martins et al. [171] assessed the antimicrobial activity and cytotoxic properties of eugenol analogs. Derivatives of eugenol were synthesized through standard acylation and alkylation methods. Most of these compounds exhibited notable antimicrobial activities. Compounds **83**–**85** in Figure 30 demonstrated the highest activity, outperforming eugenol. Compound **83** exhibited bacteriostatic activity against all four bacterial strains, including *S. aureus*, *E. coli*, *P. aeruginosa*, and *E. faecalis*. Compounds **84** and **85** displayed bactericidal effects exclusively against Gram-positive strains.

**Figure 31:**
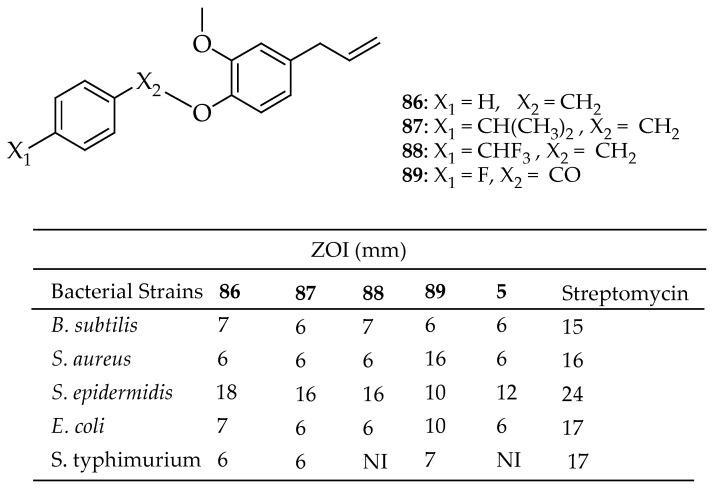
Chemical structures of compounds (**86**–**89**) and their antibacterial outcomes [173].

Regarding their antibacterial properties, substituting the hydroxy group of eugenol with an aryl group seemed to enhance the activity. Additionally, a nitro (NO_2_) group at the para position increased specificity against Gram-positive strains while showing no activity against Gram-negative strains at concentrations up to 500 μg/mL. The high antibacterial efficacy of compound **84** is likely due to the nitro functional group. These electron-withdrawing groups may reduce the electron density on the benzene ring through resonance effects, contributing to the increased activity. Moreover, consistent with these findings, Halim et al. [172] also demonstrated that nitrobenzene derivatives were active only against Gram-positive bacteria. The study by de Almeida et al. [173] validated the efficacy of eugenol derivatives **83** and **84** in Figure 31, as well as the synergistic effect of eugenol combined with rifampicin, isoniazid, ethambutol, and pyrazinamide against *Mycobacterium tuberculosis* H_37_Rv and various clinical isolates with different resistance profiles. These derivatives showed significantly higher activity against non-tuberculous mycobacteria compared to eugenol, though all compounds demonstrated low activity against other Gram-positive and Gram-negative bacteria.

Rahim et al. [173] synthesized a series of eugenol derivatives and assessed their antibacterial activity against five bacterial strains (*B. subtilis*, *S. aureus*, *S. epidermidis*, *E. coli*, and *S. typhimurium*) using a well-diffusion method. Among the tested compounds, compounds **86**–**89** in Figure 31 showed susceptibility to *S. epidermidis* with a ZOI of 16–18 mm. In comparison, compound **89** demonstrated susceptibility towards *S. aureus* with an inhibition diameter of 16 mm. The other compounds exhibited varied antibacterial activities and were classified as either intermediate or resistant, indicating that eugenol derivatives have a narrow spectrum of activity, primarily targeting Gram-positive bacteria.

**Figure 32:**
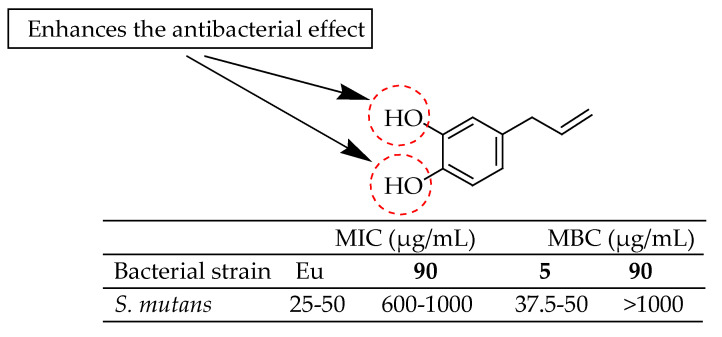
Chemical structure of compound **90** and its antibacterial outcomes [175].

Lopez et al. [174] examined the antimicrobial activity of methyl eugenol, eugenol, and hydroxychavicol against various oral bacteria. Hydroxychavicol (**90** in Figure 32) showed the lowest inhibitory and bactericidal concentrations, with MIC values ranging from 25 to 50 µg/mL and MBC values from 37.5 to 50 µg/mL. Structure-activity analysis revealed that the presence of free hydroxy groups attached to the benzene ring enhances the antibacterial effectiveness of these compounds.

**Figure 33:**
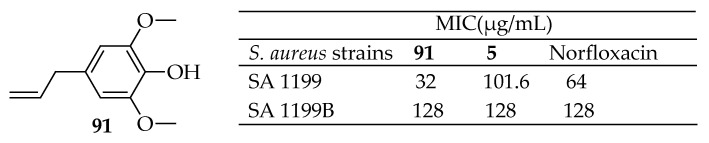
Chemical structures of compound (**91**) and its antibacterial outcomes [175].

Muniz et al. [175] investigated the antibacterial activity of eugenol and its derivatives allylbenzene, 4-allylanisole, isoeugenol, and 4-allyl-2,6-dimethoxyphenol against the *S. aureus* NorA efflux pump (EP) in combination with norfloxacin and ethidium bromide. Except for 4-allylanisole and allylbenzene, the compounds demonstrated clinically effective antibacterial activity. When combined with norfloxacin against the SA 1199B strain, 4-allyl-2,6-dimethoxyphenol, eugenol, and isoeugenol significantly reduced the MIC of the antibiotic, showing synergistic effects. Similar results were obtainable for 4-allyl-2,6-dimethoxyphenol (**91**, Figure 33), allylbenzene, and isoeugenol were used with ethidium bromide. These findings suggest that eugenol and its derivatives may inhibit the NorA pump. This in vitro evidence was supported by docking results, which showed favorable interactions between 4-allyl-2,6-dimethoxyphenol **91** and the NorA pump through hydrogen bonds and hydrophobic interactions. In conclusion, eugenol derivatives have the potential to be developed as antibacterial agents against strains carrying the NorA efflux pump.

**Figure 34:**
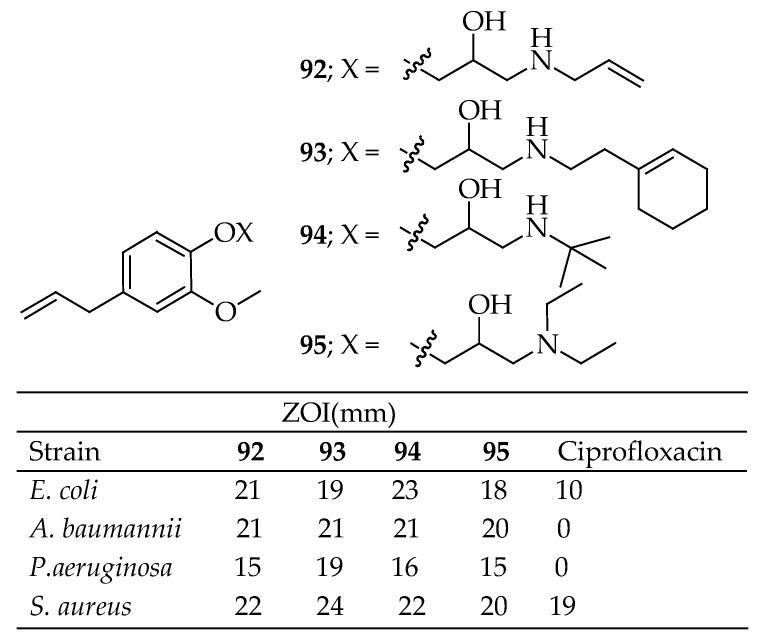
Chemical structures of compounds **92**–**95** and their antibacterial outcomes [176].

Bilgiçli et al. [176] synthesized eugenol-based oxypropanolamine derivatives with high yields, concluding that these compounds are significantly more effective than reference drugs. These compounds demonstrated strong antibacterial effects against certain MDR Gram-negative bacteria (*A. baumannii*, *P. aeruginosa*, and *E. coli*) as well as Gram-positive bacteria (*S. aureus*). Compounds such as **92**–**94** in Figure 34 exhibited similar and enhanced antibacterial activities. These eugenol-bearing oxypropanolamine derivatives also served as potent inhibitors of α-glycosidase, cytosolic carbonic anhydrase I and II isoforms (hCA I and II), and acetylcholinesterase (AChE) enzymes. The inhibition constants (Ki) were in the range of 0.80 ± 0.24–3.52 ± 1.01 μM for hCA I, 1.08 ± 0.15–3.64 ± 0.92 μM for hCA II, 5.18 ± 0.84–12.46 ± 2.08 μM for α-glycosidase, and 11.33 ± 2.83–32.81 ± 9.73 μM for AChE.

The metronidazole ether derivative of eugenol (**95**, Figure 34) was synthesized through the reaction of metronidazole tosylate with eugenol. The resulting product was evaluated for its antibacterial activity against two strains: *Helicobacter pylori* and *Clostridium perfringens* [177].

**Figure 35:**
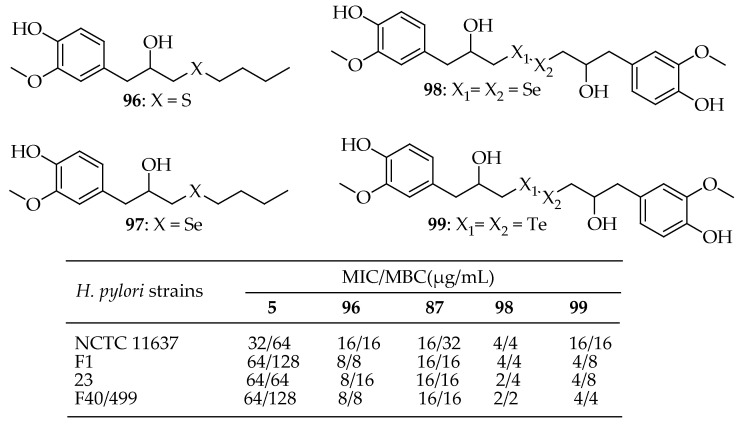
Chemical structures of compounds (**96**–**99**) and their antibacterial outcomes [178].

Carradori et al. [178] synthesized 30 compounds divided into three series (A, B, and C) and tested their antibacterial activity against four *H. pylori* strains, including a reference strain (NCTC 11637) and three drug-resistant clinical isolates (F1, 23, and F40/499). They investigated modifications around the phenolic OH group at the ortho position through aryl diazotation, O-alkylation, and O-benzylation, as well as by replacing the allyl tail and introducing one or more chalcogen atoms. Antibacterial susceptibility tests on four strains of *H. pylori*, including three clinical isolates, revealed enhanced activity in some instances. Notably, certain chalcogen-containing compounds (**96**–**99**, Figure 35) demonstrated significantly lower MIC values. For example, compounds **96**, **98**, and **99** had MICs of 16 µg/mL against the NCTC 11637 strain, while compound **96** showed a particularly low MIC of 4 µg/mL, representing a 2- to 4-fold reduction compared to EU.

However, the overall most potent compound was **98**, with MIC and MBC values ranging from 2 to 4 µg/mL across all tested strains. This compound featured a complex structure with a Se–Se bond.

**Figure 36:**
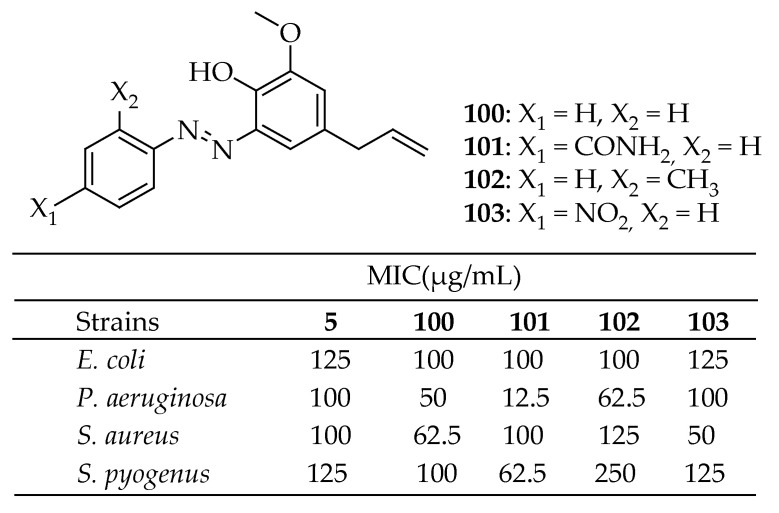
Chemical structures of compounds (**100**–**103**) and their antibacterial outcomes [179].

Dandge et al. [179] successfully synthesized five new azo dyes from the naturally occurring compound eugenol by coupling it with the diazo derivatives of various aromatic amines, achieving excellent yields. Among the newly synthesized compounds, compounds **100**–**102** in Figure 36 demonstrated exceptional antibacterial activity against the *E. coli* strain. Notably, **103** exhibited excellent antibacterial activity against the *P. aeruginosa* strain. The compound **100** (Figure 36) showed good antibacterial activity against the *S. aureus* strain, while **101** displayed good inhibitory action against *S. pyogenes*.

### 4.5. Derivatives of Cinnamaldehyde

**Figure 37:**
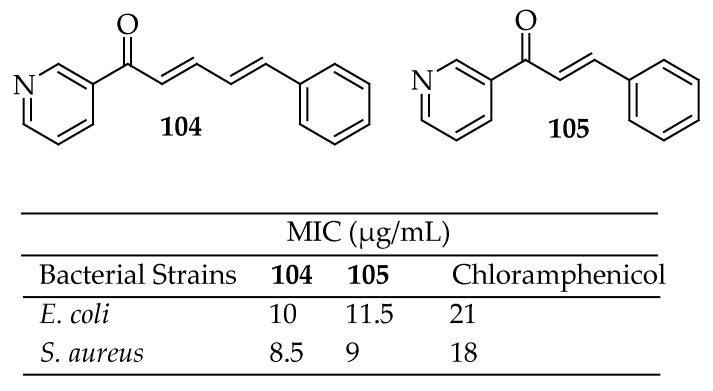
Chemical structures of compounds (**104** and **105**) and their antibacterial outcomes [185].

Cinnamaldehyde constitutes approximately 90% of the EO from cinnamon bark [180]. While various laboratory synthetic methods exist, the steam distillation of cinnamon bark oil yields the most cost-effective cinnamaldehyde. The FDA has recognized cinnamaldehyde as GRAS. The European legislation has approved it for consumer products [181]. Cinnamaldehyde’s antimicrobial activity is attributed to multiple mechanisms, some of which are interrelated. Studies on its bactericidal effects against *L. monocytogenes* and *E. coli* suggest that its interaction with the cell membrane rapidly inhibits energy metabolism. This disruption of the proton motive force causes the leakage of small ions without affecting more significant components like ATP, along with the inhibition of ATP production and membrane-bound adenosine triphosphatase (ATPase) activity [182,183]. In 2019, Doyle et al. [184] summarized the existing knowledge on the antibacterial properties and mechanisms of cinnamaldehyde and its derivatives, emphasizing notable advancements in this field of research. They concluded that these compounds and their mechanisms of action merit additional investigation as potential antibacterial agents and targets. However, the current understanding of the antibacterial mechanisms of cinnamaldehyde derivatives, in particular, is limited. Consequently, further research is needed to optimize the potential of these compounds and targets. Recently, Amaliyah et al. [185] synthesized two azachalcone derivatives **104** and **105** (Figure 37) from cinnamaldehyde and evaluated their antibacterial activity. The results demonstrated significantly enhanced antibacterial activities.

**Figure 38:**
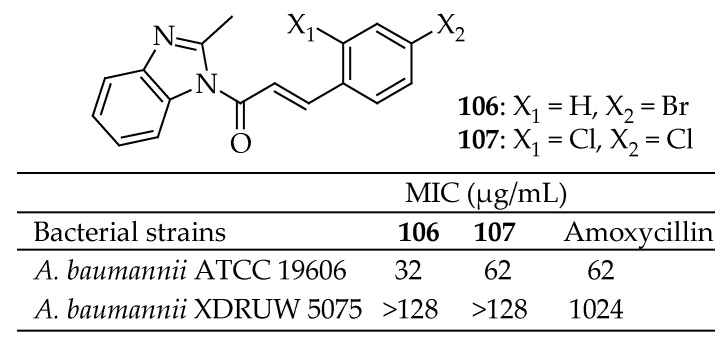
Chemical structures of compounds (**106** and **107**) with their antibacterial outcomes [110].

Chai et al. [110] investigated the antimicrobial effectiveness, target-specific activity, and pre-clinical viability of a series of 2-methylbenzimidazoyl cinnamaldehyde analogs. Six compounds exhibited antibacterial activity against the critical priority pathogen *A. baumannii*, with the 4-bromophenyl-substituted compound **106** in Figure 38 showing the most potent antimicrobial activity (MIC 32 μg/mL). These compounds inhibited cell division in *A. baumannii*, indicated by an elongated phenotype, and targeted the FtsZ protein by inhibiting its polymerization and GTPase activity. In silico docking suggested that the compounds bind in the interdomain cleft near the H7 core helix. These findings identify halogenated analogs **106** and **107** as promising candidates for further development as antimicrobial agents against *A. baumannii*.

**Figure 39:**
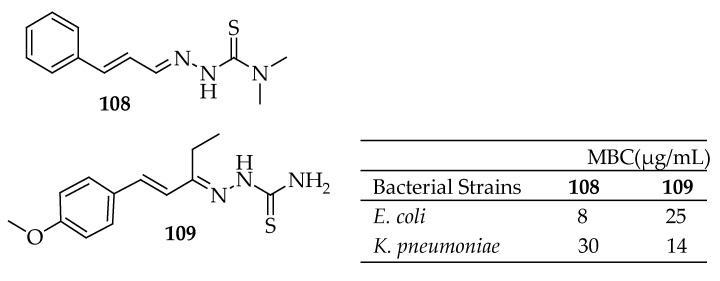
Chemical structures of compounds (**108** and **109**) with antibacterial outcomes [186].

Bisceglie et al. [186] developed nine metal complexes based on cinnamaldehyde thiosemicarbazone analogues and evaluated their antibacterial activities. Out of the nine synthesized molecules, only compounds **108** and **109**, shown in Figure 39, demonstrated inhibitory or bactericidal activity against the tested strains. Compound **108** exhibited the highest activity against *E. coli* with an MBC of 8 μg/mL, while compound **109** was most effective against *K. pneumoniae*, with an MBC of 14 μg/mL. Other compounds displayed no activity at concentrations up to 50 μg/mL.

**Figure 40:**
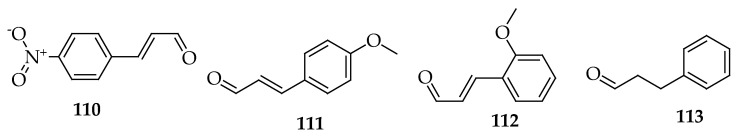
Chemical structures of compounds **110**–**113 [187]**.

Van Liefferinge et al. [187] assessed the in vitro and in vivo antimicrobial activity of cinnamaldehyde and its derivatives **110**–**113** (Figure 40) against the intestinal bacteria of weaned piglets (*Coliform* bacteria, *Enterococci*, *Lactobacilli*, and *anaerobic* bacteria). In vitro results revealed that 4-nitrocinnamaldehyde (**110**) exhibited the highest antimicrobial activity, but due to its carcinogenic properties, it was not pursued further. Cinnamaldehyde displayed the second highest activity, particularly against *coliform* bacteria and *E. coli*, followed by 4-methoxycinnamaldehyde (**111**), 2-methoxycinnamaldehyde (**112**), and hydrocinnamaldehyde (**113**). Other derivatives displayed lower potency but were more effective against *coliform* bacteria and *E. coli* than the Gram-positive bacteria. At pH 7, aldehydes demonstrated greater bactericidal activity than their corresponding carboxylic acids, though this was not observed at pH 5, suggesting a different mode of action. In the in vivo trial, no significant improvements in animal performance or antimicrobial effects were visible. Overall, except for 4-nitrocinnamaldehyde (**110**), none of the derivatives showed superior antimicrobial potency compared to cinnamaldehyde, and three compounds that showed promise in vitro did not yield significant positive outcomes in vivo.

## 5. Conclusions

EO components such as carvacrol, thymol, menthol, cinnamaldehyde, and eugenol have demonstrated significant antibacterial properties against pathogenic microorganisms. Their efficacy, which is influenced by their chemical structures, mechanisms of action, and interaction with bacterial cell walls and membranes, reveals them as alternative or complementary therapies to conventional antibiotics. The associated challenges, such as limited bioavailability, stability, and potential toxicity at higher concentrations, need further investigation [188]. Structural modifications, such as the synthesis of derivatives, have shown potential in enhancing their antibacterial efficacy, suggesting a valuable area for future research. Among the reported EO components, compounds like thymol, carvacrol, and their derivatives demonstrated potent antibacterial activity, often comparable to, or surpassing, conventional antibiotics. For instance, derivatives modified with phenolic groups generally showed enhanced efficacy, suggesting that targeted structural modifications may further optimize their antibacterial effects. Key factors that increase their antibacterial activity include the reactive groups (e.g., phenolic hydroxy and aldehyde), unsaturated bonds, optimal alkyl chain length, and halogenation, which improve membrane interaction. In contrast, masking critical functional groups and excessive modifications can reduce effectiveness. Additionally, synergistic modifications, such as combining these components with antibiotics or bioactive moieties, can enhance antibacterial potency.

These findings demonstrate the potential of EO components as valuable therapeutic agents for developing effective antibacterial agents, particularly in combatting MDR strains. However, further studies are needed to fully understand the mechanisms of action, improve stability and bioavailability, and evaluate their safety in clinical settings. By addressing these gaps, future research will advance these natural products toward practical therapeutic applications.
